# Recycling of the major thylakoid lipid MGDG and its role in lipid homeostasis in *Chlamydomonas reinhardtii*

**DOI:** 10.1093/plphys/kiab340

**Published:** 2021-07-23

**Authors:** Masako Iwai, Yui Yamada-Oshima, Kota Asami, Takashi Kanamori, Hideya Yuasa, Mie Shimojima, Hiroyuki Ohta

**Affiliations:** School of Life Science and Technology, Tokyo Institute of Technology, Yokohama 226-8501, Japan

## Abstract

Monogalactosyldiacylglycerol (MGDG), the most abundant lipid in thylakoid membranes, is involved in photosynthesis and chloroplast development. MGDG lipase has an important role in lipid remodeling in *Chlamydomonas reinhardtii*. However, the process related to turnover of the lysogalactolipid that results from MGDG degradation, monogalactosylmonoacylglycerol (MGMG), remains to be clarified. Here we identified a homolog of *Arabidopsis thaliana* lysophosphatidylcholine acyltransferase (LPCAT) and characterized two independent knockdown (KD) alleles in *C. reinhardtii*. The enzyme designated as *C. reinhardtii*Lysolipid Acyltransferase 1 (CrLAT1) has a conserved membrane-bound *O*-acyl transferase domain. LPCAT from Arabidopsis has a key role in deacylation of phosphatidylcholine (PC). *Chlamydomonas reinhardtii*, however, lacks PC, and thus we hypothesized that CrLAT1 has some other important function in major lipid flow in this organism. In the CrLAT1 KD mutants, the amount of MGMG was increased, but triacylglycerols (TAGs) were decreased. The proportion of more saturated 18:1 (9) MGDG was lower in the KD mutants than in their parental strain, CC-4533. In contrast, the proportion of MGMG has decreased in the *CrLAT1* overexpression (OE) mutants, and the proportion of 18:1 (9) MGDG was higher in the OE mutants than in the empty vector control cells. Thus, CrLAT1 is involved in the recycling of MGDG in the chloroplast and maintains lipid homeostasis in *C. reinhardtii*.

## Teaser

A recycling system of the major thylakoid lipid monogalactosyldiacylglycerol in the *Chlamydomonas reinhardtii* chloroplast contributes to lipid homeostasis.

## Introduction

Monogalactosyldiacylglycerol (MGDG) is the most abundant lipid in the photosynthetic membranes called thylakoids in cyanobacteria and chloroplasts of algae and plants and is involved in both photosynthesis and chloroplast development (Block et al., 1983; Shimojima et al., 1997; Kobayashi et al., 2009a; Shimojima and Ohta, 2011; Boudière et al., 2014; Kalisch et al., 2016). In land plants, the last step in MGDG synthesis occurs in plastid envelope membranes. This reaction is catalyzed by MGDG synthase, which transfers a galactosyl residue from UDP-galactose to the *sn*-3 position of *sn*-1,2-diacylglycerol (DAG; Shimojima et al., 1997; Benning and Ohta, 2005; Shimojima and Ohta, 2011). MGDG is the precursor for the biosynthesis of another major galactolipid, digalactosyldiacylglycerol (DGDG; Dörmann et al., 1999). Thus, MGDG is not only the main constituent of thylakoid membranes but also a substrate for DGDG synthesis.

In *Arabidopsis thaliana*, three functional MGDG synthases (MGD1, MGD2, and MGD3) have been identified and classified into type-A (MGD1) and type-B (MGD2 and MGD3) enzymes based on their amino acid identity (Awai et al., 2001). The type-A enzyme is responsible for the bulk of MGDG biosynthesis. Type-B enzymes are responsible for P starvation-induced galactolipid accumulation (Awai et al., 2001; Kobayashi et al., 2004, 2009b).

The unicellular eukaryotic alga *Chlamydomonas reinhardtii* contains one large chloroplast in its cell and has an enriched amount of MGDG among its total membrane lipids as compared with land plants (Giroud et al., 1988; Li-Beisson et al., 2013). In *C. reinhardtii*, MGDG synthase (MGD1) has also been identified, and the expression of *MGD1* is downregulated under P starvation (Hidayati et al., 2019).

Although MGDG biosynthesis has been well characterized, the catabolic process of MGDG has not been studied in detail. Two gene families have been reported to encode MGDG lipases in land plants: DEFECTIVE IN ANTHER DEHISCENCE1-like proteins (Ishiguro et al., 2001) and patatin-like lipid acyl hydrolases (Matos et al., 2001, 2008). HEAT INDUCIBLE LIPASE1 (HIL1) has an important role in the lipid remodeling process induced by heat stress in Arabidopsis leaves (Higashi et al., 2018). HIL1 encodes a MGDG lipase that is localized in chloroplasts and releases 18:3-free fatty acid (FA) in the first committed step of 34:6 (18:3/16:3)-containing MGDG turnover. SENSITIVE TO FREEZING2 (SFR2), a galactolipid:galactolipid galactosyltransferase (GGGT), contributes to lipid remodeling during freezing stress to enhance freezing tolerance in Arabidopsis (Moellering et al., 2010). In *C. reinhardtii*, although there is no SFR2 homolog or GGGT activity (Fan et al., 2011; Warakanont et al., 2015), phospholipid:DAG acyltransferase (CrPDAT) acts as a galactolipid:DAG acyltransferase, transferring a fatty acyl group from MGDG to DAG to form triacylglycerol (TAG; Yoon et al., 2012). CrPDAT uses chloroplast membrane lipids, particularly MGDG, sulfoquinovosyldiacylglycerol (SQDG), and phosphatidylglycerol (PG), as substrates to synthesize TAG in vivo. Chlamydomonas PLASTID GALACTOGLYCEROLIPID DEGRADATION1 (PGD1) is a MGDG-specific lipase (Li et al., 2012). PGD1 plays essential role in maintaining the appropriate thylakoid membrane composition and structure (Du et al., 2018). PGD1 acts predominantly on more saturated MGDG and specifically on MGDG assembled from de novo-synthesized FAs (18:1 (9)/16:0) under N-deficient conditions (Li et al., 2012). *Chlamydomonas reinhardtii* accumulates substantial amounts of TAGs under stress conditions (Wang et al., 2009; Li et al., 2010; Work et al., 2010; Iwai et al., 2014; Légeret et al., 2016). Galactoglycerolipid represents a major source of FAs esterified in TAGs following N deprivation. The *pgd1* mutant has altered MGDG abundance and acyl composition and altered abundance of photosynthesis complexes, with an increased photosystem II/photosystem I ratio (Du et al., 2018). These MGDG lipases preferentially release the *sn*-1 acyl groups of MGDG (Li et al., 2012; Higashi et al., 2018). It is not clear yet whether lysogalactolipid (monogalactosylmonoacylglycerol [MGMG]), which results from this digestion of MGDG, contributes as a substrate for recycling of MGDG or is hydrolyzed. We hypothesized that some unidentified gene(s) that encodes an acyltransferase and is induced by stress conditions could participate in the acylation of MGMG, leading to recycling of MGDG.Phosphatidylcholine (PC) is one of the most rapidly labeled and metabolized membrane lipids in seed plants, and acyl exchange involving PC has been suggested to play a role in the export of FAs relevant for extraplastidic lipid biosynthesis, including that of TAGs (Bates et al., 2007, 2009). In budding yeast (*Saccharomyces cerevisiae*), yeast oligomycin resitance 175C (*YOR175C*), also known as acyltransferase for lyso-PtdEtn 1 (*ALE1*), sphingolipid compensation 4, or acetyl-CoA:lyso-platelet-activating factor acetyltranferase 1, encodes an acyl-CoA-dependent lysophospholipid acyltransferase (LPLAT) that can acylate a wide range of substrates including lysophosphatidic acid, lysophosphatidylcholine (LPC), lysophosphatidylethanolamine, lysophosphatidylglycerol, lysophosphatidylinositol, and lysophosphatidylserine (Benghezal et al., 2007; Chen et al., 2007a; Jain et al., 2007; Riekhof et al., 2007; Tamaki et al., 2007). ScLPLAT belongs to a distinct subfamily of membrane-bound *O*-acyl transferase (MBOAT) proteins (Hofmann, 2000). Genes in the MBOAT family are widely distributed from *S. cerevisiae* to humans. The MBOAT family is composed of gene members encoding a variety of acyltransferase enzymes, which play important roles in plant acyl lipid metabolism. In Arabidopsis, acyl-CoA:DAG acyltransferase (DGAT), acyl-CoA:lysophosphatidylcholine acyltransferase (LPCAT), and acyl-CoA sterol acyl transferase are MBOAT family members (Hobbs et al., 1999; Chen et al., 2007b; Ståhl et al., 2008). LPCATs play key roles in the Lands cycle (Lands, 1958; Ståhl et al., 2008; Shindou and Shimizu, 2009) in which they are involved in a deacylation–reacylation process that starts with the deacylation of PC to produce LPC, followed by a reaction that reacylates LPC to PC (Stymne and Stobart, 1984). PC serves as the acyl donor during the formation of TAG from DAG, which is a precursor and a building block for TAG biosynthesis (Moessinger et al., 2014; Zulu et al., 2018). In Arabidopsis, LPCAT1(At1g12640) and LPCAT2(At1g63050) participate in the Lands cycle in developing seeds (Wang et al., 2012). When DGAT1(At2g19450) is eliminated, enhanced LPCAT2 activity supplies the additional PC required for PDAT1(At5g13640) in Arabidopsis TAG synthesis (Xu et al., 2012). PC acyl editing and phosphocholine headgroup exchange between PC and DAG control most of the acyl flux through PC to provide polyunsaturated FAs for TAG synthesis in Arabidopsis seeds (Bates et al., 2012). Although most eukaryotic organisms contain PC, *C. reinhardtii* does not (Sato and Furuya, 1985; Giroud et al., 1988). To reveal the flow of lipid biosynthesis in *C. reinhardtii*, we focused on MGMG produced in *C. reinhardtii* and isolated a LPLAT (ScALE1) homolog, which we named *C. reinhardtii*Lysolipid Acyltransferase 1 (CrLAT1). Our findings suggest that CrLAT1 is involved in the recycling of MGDG and contributes to the maintenance of lipid homeostasis in *C. reinhardtii*.

## Results

### Identification of MGMG in *C. reinhardtii*

**Figure 1:**
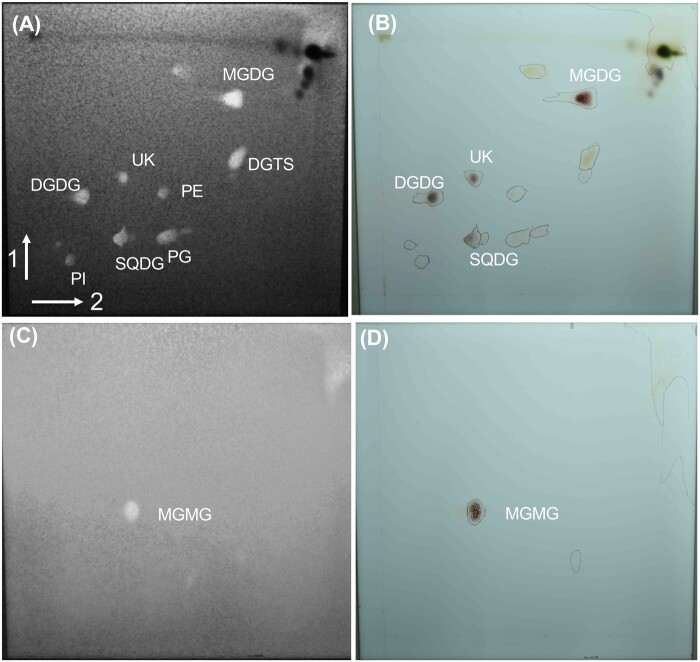
Fractionation by 2D-TLC of total lipids from cells of *C. reinhardtii* CC-4533 grown in TAP medium. Chromatography was performed in chloroform/methanol/water (65:25:4, v/v/v) for the first dimension (arrow 1) and chloroform/methanol/isopropylamine/ammonia water (65:35:0.5:5, v/v/v/v) for the second dimension (arrow 2). A and B, Total lipid from cells of *C. reinhardtii* CC-4533. C and D, MGMG from the enzymatic hydrolysis of *C. reinhardtii* CC-4533 MGDG by *R. arrhizus* lipase. A and C, Primuline staining of all lipids. Bright fluorescent spots indicate lipids. Dark spots indicate pigments. B and D, Galactolipid staining with anthrone–sulfuric acid reagent. PE, phosphatidylethanolamine; PI, phosphatidylinositol.

**Figure 2:**
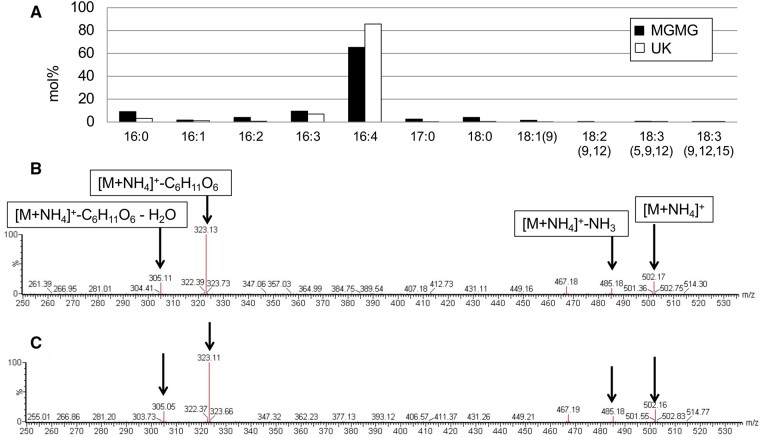
FA profile and fragmentation spectra of MGMG and the UK. A, FA profile of the UK and MGMG. MGMG was produced by digestion of MGDG isolated from *R. arrhizus* by a lipase. MGMG and the UK were separated by TLC before GC–FID. Fragmentation spectra for representative MGMG (B) and the UK (C) species were obtained from *C. reinhardtii*. For each major ion, the *m*/*z* value and molecular species (black arrows) are indicated.

**Figure 3.**
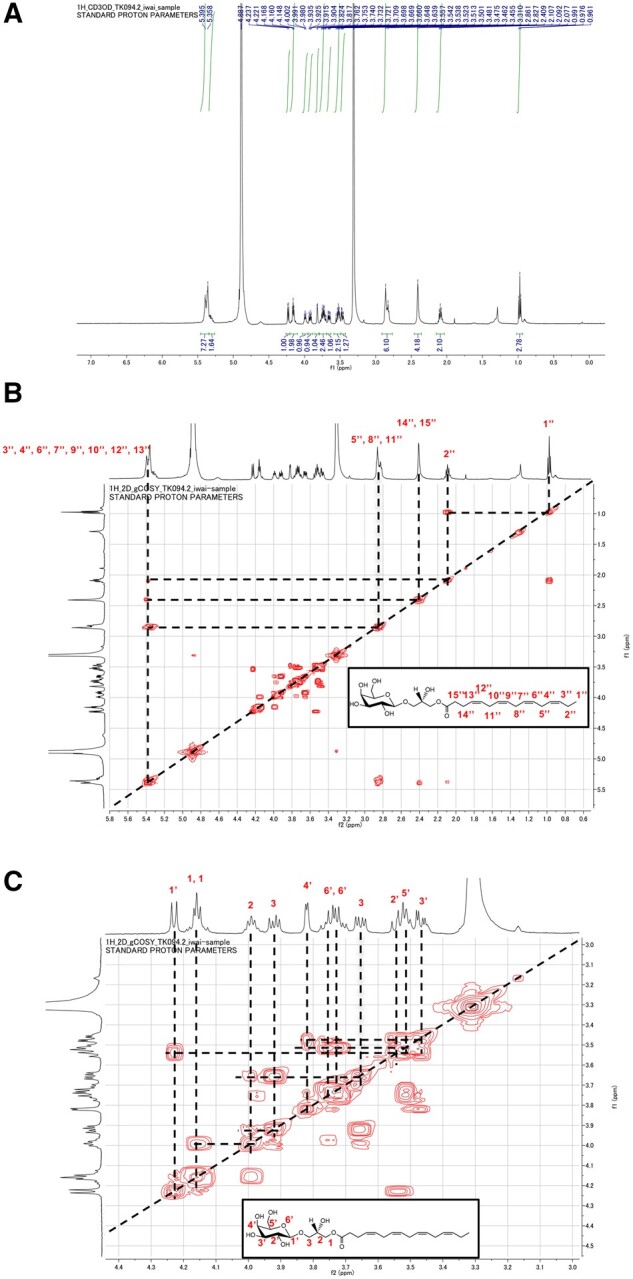
NMR spectrum of the UK. ^1^H NMR spectrum (A), full ^1^H correlation spectroscopy (COSY) NMR chart (B), and the expanded COSY chart (C) for the UK as measured in CD_3_OD. The cross peaks except for the diagonal peaks in the COSY charts indicate couplings between pairs of ^1^H peaks.

*Chlamydomonas reinhardtii* CC-4533 (*cw15*, mt^−^) cells grown in Tris–acetate–phosphate (TAP) medium were harvested, and their lipids were extracted and separated by 2D thin-layer chromatography (2D-TLC). An unknown lipid (UK) was stained reddish purple with the anthrone–sulfuric acid reagent, which is commonly used for the detection of sugar-containing lipids (Radin et al., 1955; Figure 1, A and B). The UK was also detected in total lipid extracted from another wild-type (WT) strain, CC-125 (mt^+^; Supplemental Figure S1). The UK was recovered from the plate, and its FA profile was quantified by gas chromatography–flame ionization detection (GC–FID). Surprisingly, the major FA in the UK was C16:4 (Figure 2A). C16:4 is mainly found in the *sn*-2 position of MGDG, which is synthesized exclusively by the chloroplast (Giroud et al., 1988; Sato, 1989). We hypothesized that the UK is the lysogalactolipid MGMG and compared the FA profiles of the UK with MGMG from the enzymatic hydrolysis of MGDG. MGDG isolated from total lipids of *C. reinhardtii* CC-4533 was digested by *Rhizopus arrhizus* lipase, which acts specifically on the *sn*-1 position of glycolipids (Fischer et al., 1973). MGMG was separated by TLC before GC–FID (Figure 1, C and D). The 2D-TLC spot corresponding to MGMG and the FA profile of MGMG were similar to those of the UK (Figures 1 and 2A). To investigate whether the UK is MGMG, we first determined the chemical structure of the UK with an ultra-performance liquid chromatography coupled with a tandem quadrupole mass spectrometer equipped with an electrospray ionization (UPLC–ESI–qMS/MS) system. The mass spectrum of the C16:4-MGMG is shown in Figure 2B. C16:4-MGMG (molecular weight of 484) was detected as the [M+NH_4_]^+^ ion at *m/z* 502 and exhibited a major fragment ion at *m*/*z* 323 in product ion scan mode. The loss of the glycosyl group, the polar head group (179 *m/z*), yielded monoacylglycerol with 16:4 FA (323 *m/z*). The mass spectrum of the UK (Figure 2C) is quite similar to that of C16:4-MGMG. To corroborate the identity of the UK, we next analyzed the lipid by high-resolution proton nuclear magnetic resonance (NMR) spectroscopy. The NMR spectrum of the UK corresponded with (2*S*)-1-*O*-hexadeca-4,7,10,13-tetraenoyl-3-*O*-β-d-monogalactopyranosyl glycerol (Figure 3). The UK is MGMG, in which the C16:4 acyl chain is bound to the *sn*-1 position of glycerol. The signals were identical to *sn*-1 acylglycerol reported previously (Rho et al., 1997). It is well known that polyunsaturated C16 FAs are mostly esterified at the *sn*-2 position in MGDG. However, we could not detect the *sn*-2 form. *sn*-2-MGMG is unstable and easily converted to *sn*-1-MGMG, particularly under acidic or basic conditions. As the UK was separated under a basic condition (the second dimension of TLC) before NMR, most acyl groups probably migrated to the *sn*-1 position. Although it cannot be denied that the UK is *sn*-1 acylglycerol or a mixture of *sn*-1 acylglycerol and *sn*-2 acylglycerol in the living cells, these data indicated that the UK in Figure 1 is MGMG, and thus MGMG accumulates in *C. reinhardtii* cells grown in TAP medium.

## CrLAT1 has a conserved MBOAT domain

**Figure 4:**
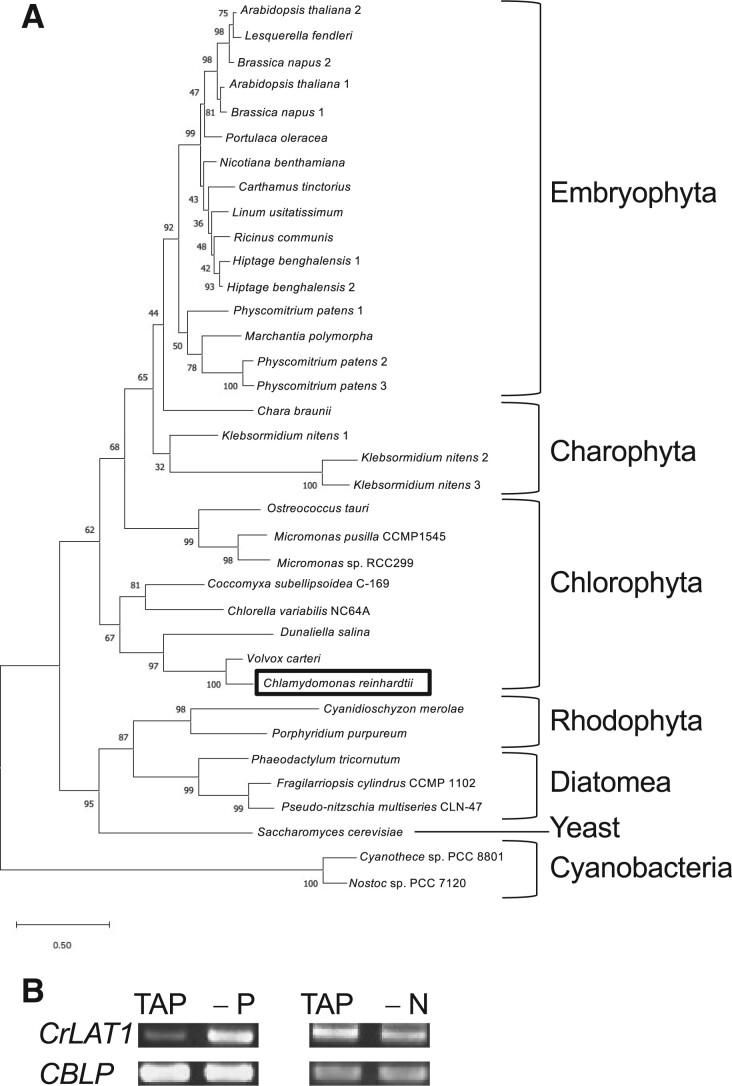
Phylogenetic tree analysis of LPCAT1 homologs and expression of CrLAT1 in *C. reinhardtii*. A, A phylogenetic tree was constructed using the maximum likelihood algorithm to compare protein sequences and to calculate topologies and branch lengths. The tree is drawn to scale, with branch lengths indicating the number of substitutions per site. Sequence accession numbers or sequence resources for the individual members of the tree are as follows: *A. thaliana* 1 (At1g12640), *A. thaliana* 2 (At1g63050), *Brassica napus* 1 (XP_013742603), *Brassica napus* 2 (XP_013661594.1), *Carthamus tinctorius* (KC763798), *Chara braunii* (95g00920), *C. reinhardtii* (Cre12.g537641), *Chlorella variabilis* NC64A (20761), *Coccomyxa subellipsoidea* C-169 (27778), *Cyanidioschyzon merolae* (CMI139C), *Cyanothece* sp. PCC 8801 (643473146), *Dunaliella salina* (0546s00001), *Fragilarriopsis cylindrus* CCMP 1102 (208289), *Hiptage benghalensis* 1 (KC763795), *Hiptage benghalensis* 2 (KC763797), *Klebsormidium nitens* 1 (kfl00025_0100), *Klebsormidium nitens* 2 (kfl00029_0360), *Klebsormidium nitens* 3 (kfl00029_0290), *Lesquerella fendleri* (KC667073), *Linum usitatissimum* (10006325), *M. polymorpha* (0004s0125), *Micromonas pusilla* CCMP1545 (23556), *Micromonas* sp. RCC299 (93794), *Nicotiana benthamiana* (101Scf02368g00011), *Nostoc* sp. PCC 7120 (637234453), *Ostreococcus tauri* (ostta18g00810), *Phaeodactylum tricornutum* (Phatr2_49702), *Physcomitrium (Physcomitrella) patens* 1 (3c6_27300), *Physcomitrium (Physcomitrella) patens* 2 (3c11_9320), *Physcomitrium (Physcomitrella) patens* 3 (3c7_18311), *Porphyridium purpureum* (2296.16), *P. oleracea* (MG551550), *Pseudo-nitzschia multiseries* CLN-47 (283768), *Ricinus communis* (W8DT11), *S. cerevisiae* (YOR175c), and *V. carteri* (0034s0011). B, Expression of *CrLAT1* in *C. reinhardtii* under TAP, TAP −P (−P) or TAP −N (−N) conditions. *CBLP* served as a control.

The MGDG-specific lipase PGD1 is present in *C. reinhardtii* (Li et al., 2012) and another MGDG lipase HIL1 is present in Arabidopsis (Higashi et al., 2018). We believe that there is a reacylation step related to MGMG, because MGDG is a major component of thylakoid membranes in the *C. reinhardtii* chloroplast, and its presence is important for maintaining chloroplast function (Giroud et al., 1988; Boyle and Morgan, 2009). To identify the molecular components of the MGMG reacylation step, we searched for possible acyl transferase genes in the *C. reinhardtii* genome database at Phytozome version 12 using BLAST searches for homologous proteins and also for the conserved MBOAT domain. We found a putative acyl transferase, Cre12.g537641. The predicted amino acid sequence of Cre12.g537641, which we named CrLAT1, contains the MBOAT domain. ChloroP and PredAlgo algorithms suggested that CrLAT1 does not have a chloroplast transit peptide. The TargetP-2.0 algorithm suggested that CrLAT1 has neither a signal peptide nor a transit peptide (Supplemental Table S1). A phylogenetic tree was generated using the predicted amino acid sequence from CrLAT1 and the homologous proteins from other species (Figure 4A). LPCATs from land plants in the phylogenetic tree can acylate lysophospholipids when incubated in vitro with these substrates and ^14^C-labeled acyl-CoA (Ståhl et al., 2008; Zheng et al., 2012; Lager et al., 2013; Zhang et al., 2015; Jasieniecka-Gazarkiewicz et al., 2016). LPCATs from the genomes of *Physcomitrium (Physcomitrella) patens*, *Marchantia polymorpha*, Charophyta, and algae have been identified, but their functions have not yet been confirmed. Phylogenetic analysis showed that CrLAT1 belongs to a Chlorophyta subfamily. As shown in Figure 4B, expression of *CrLAT1* was increased in *C. reinhardtii* under P depletion but was not increased after N depletion. After P depletion, *CrMGD1* showed decreased expression based on RNA sequencing (RNA-seq; Hidayati et al., 2019), whereas *CrLAT1* showed increased expression in WT strains (Supplemental Table S2).

We examined gene coexpression analyses on the ATTED-II website (http://atted.jp, version 9.2; Obayashi et al., 2018; Supplemental Tables S3 and S4) and the ALCOdb website (http://alcodb.jp; Aoki et al., 2016; Supplemental Table S5). *AtLPCAT1* was coexpressed with the lipid biosynthesis genes Lipid-binding serum glycoprotein family protein, LPS-binding protein and bactericidal/permeability-increasing protein-related protein 1 (At1g04970), serinc-domain containing serine and sphingolipid biosynthesis protein (At1g16180) and Calcium-dependent lipid-binding domain family protein, C2-domain abscisic acid -related protein 10 (At2g01540). *AtLPCAT2* was coexpressed with ribosomal genes. *CrLAT1* was coexpressed with the diacylglyceryltrimethylhomoserine (DGTS) biosynthesis gene, betaine lipid synthase 1 (*BTA1*; Cre07.g324200), and the SQDG biosynthesis gene *SQD1* (Cre16.g656400). The proportions of DGTS and SQDG both increase in *C. reinhardtii* under P-depleted conditions (Hidayati et al., 2019). Consistently, *BTA1* and *SQD1* show increased expression in response to P depletion in *C. reinhardtii* (Hidayati et al., 2019). This suggested that *CrLAT1* and the coexpressed genes coordinately played a role in the remodeling of membrane lipids during P depletion.

## MGMG was increased in *CrLAT1* knockdown mutants

**Figure 5:**
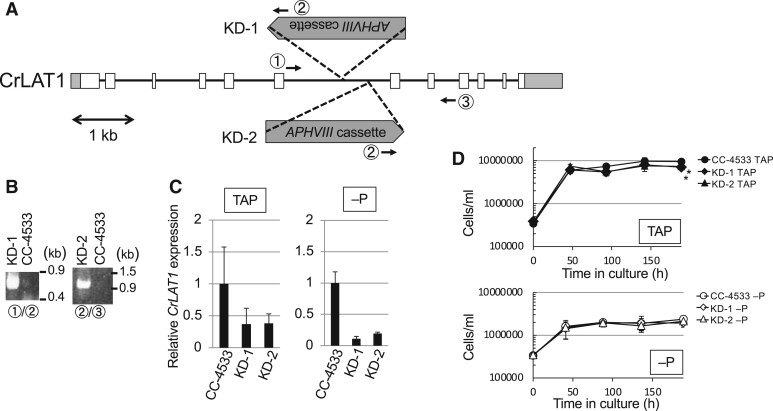
Isolation and characterization of *C. reinhardtii* KD-1 and KD-2 mutants from CLiP. A, Schematic representation of insertion sites of the *APHVIII* cassette in the genomic sequence of Cre12.g537641. Gray and white boxes indicate untranslated regions and protein-coding regions, respectively. Arrows indicate the location of primers 1–3 used to detect *APHVIII* cassette insertions in (B). B, Genotyping of the KD-1 and KD-2 mutants. Genomic DNA fragments were amplified by PCR using the primer sets indicated in (A). C, KD-1 and KD-2 suppressed the expression of *CrLAT1*. Cells were cultured for 5 d under TAP and TAP −P (−P) conditions, and then *CrLAT1* mRNA was assayed using RT-qPCR. The values were normalized to the expression of *CBLP* (G protein beta subunit-like polypeptide). Error bars represent standard errors based on four biological replicates. D, Growth of *C. reinhardtii* CC-4533 and mutants grown in TAP medium or under P starvation. *Chlamydomonas reinhardtii* cells precultured to logarithmic phase in TAP medium were then inoculated into TAP or TAP –P (−P) medium. Values represent the mean ± sd from four independent replicates. Asterisks indicate a statistically significant difference compared with CC-4533 based on a two-tailed Student’s *t* test (^*^P < 0.05).

To investigate the function of CrLAT1 in vivo, we used knockdown (KD) mutants (KD-1, KD-2) from the Chlamydomonas Library Project (CLiP; https://www.chlamylibrary.org). Figure 5A summarizes the antibiotic resistance gene insertion sites of the KD-1 and KD-2 mutants, which were identified by PCR (Figure 5B) and DNA sequence analysis. KD-1 and KD-2 contain an *APHVIII* DNA fragment inserted into the sixth intron of *CrLAT1*. For the two mutants, the level of *CrLAT1* transcripts in cells grown in TAP and TAP −P medium as measured by reverse transcription-quantitative PCR (RT-qPCR) was reduced to ∼30% and 10%, respectively, of that in the parental CC-4533 cells (Figure 5C). KD-1 and KD-2 cells showed slower growth than the parental CC-4533 cells in TAP medium (Figure 5D). When cultivated under P-deprived conditions, KD-1 and KD-2 cells were not significantly different in their growth rates relative to that of CC-4533 cells. These findings suggested that CrLAT1 KD influenced cell physiology in TAP medium.

**Figure 6:**
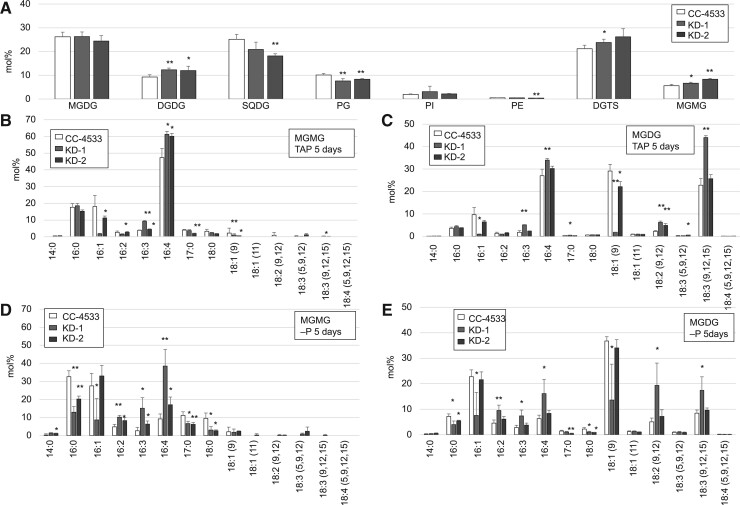
Analyses of major lipid classes and FA composition of MGMG and MGDG in *C. reinhardtii* CC-4533 and the CrLAT1 KD mutants. Cells were cultured in TAP medium and in TAP –P medium for 5 d. Values are the mean ± sd from four independent experiments. Asterisks indicate a statistically significant difference compared with CC-4533 based on a two-tailed Student’s *t* test (^*^P < 0.05 and ^**^P < 0.01). A, Relative abundance of major lipid classes of *C. reinhardtii* CC-4533 and the CrLAT1 KD mutants in TAP medium. Analyses of FA composition of MGMG (B, D) and MGDG (C, E) in *C. reinhardtii* CC-4533 and the *CrLAT1* KD mutants in TAP medium (B, C) and in TAP –P medium (D, E).

To test the effect of *CrLAT1* KD, we cultured KD-1 and KD-2 in TAP medium and analyzed their lipid content. The MGMG molar ratio was higher in KD-1 and KD-2 cells than in CC-4533 cells (Figure 6A). The ratio of MGDG did not show significant differences between CC-4533 and KD lines, but that of DGDG increased 2%–3%, indicating that the balance between MGDG and DGDG was shifted toward DGDG. The major MGMG FAs in CC-4533, KD-1, and KD-2 cells were 16:4 (Figure 6B) in TAP medium. With respect to MGMG FAs, the proportion of palmitoleic (16:1 ω-7) FA was lower, whereas that of 16:4 was higher in KD-1 and KD-2 relative to CC-4533 (Figure 6B). In the case of MGDG FAs, the major FAs in CC-4533, KD-1, and KD-2 cells were 16:4 and alpha-linolenic acid (18:3 n3). The proportions of 16:1 and oleic (18:1 ω-9) FA were lower, whereas those of 16:4, 18:3, and 18:2 were higher in KD-1 MGDG than in CC-4533 MGDG. The proportion of 18:1 (9) was lower than that in CC-4533, whereas that of 18:2 was higher in KD-2 MGDG. These showed that the ratio of mature MGDG including more polyunsaturated FA was increased in KD-1 and KD-2 (Figure 6C;Supplemental Figure S2).

**Figure 7:**
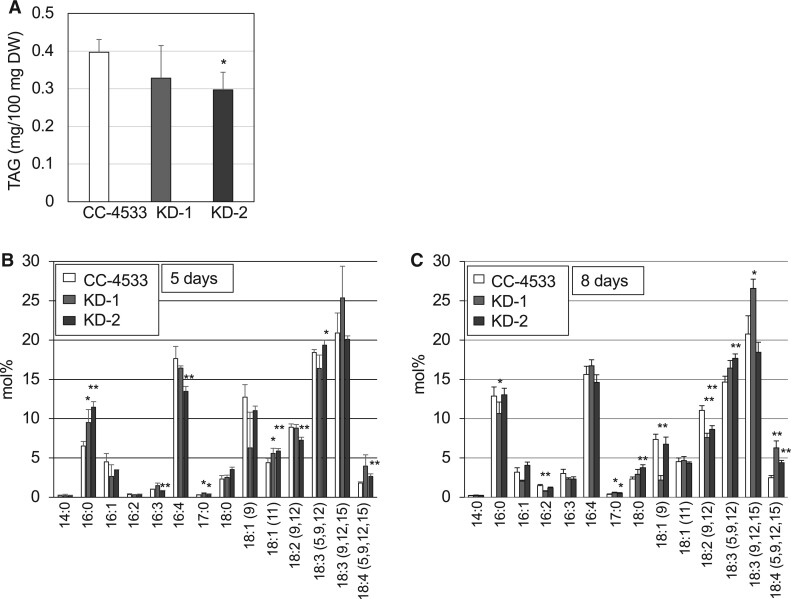
Changes in the TAG content of *C. reinhardtii* CC-4533 and the *CrLAT1* KD mutants. A, Total TAG per dry weight (DW) of cells. B and C, FA composition of the TAG fraction in CC-4533 and the mutants. Cells were cultured in TAP medium for 5 d (A, B) or 8 d (C). Values are the mean ± sd from four independent experiments. Asterisks indicate a statistically significant difference as compared with CC-4533 cells based on a two-tailed Student’s *t* test (^*^P < 0.05 and ^**^P < 0.01).

We investigated whether TAGs were differentially accumulated in KD-1 and KD-2 cells and CC-4533 cells. The level of TAGs in KD-2 cells was lower than that in CC-4533 cells (Figure 7A), similar to the finding in the *pgd1* mutant, although the TAG level in KD-1 was not significantly different from that of CC-4533. Within the TAG fraction, KD-1 and KD-2 showed a similar trend that the proportion of 18:4, the most unsaturated FA with a chain length of 18 carbon atoms was higher in the mutants than in CC-4533 (Figure 7, B and C). When cells were grown in P-deficient medium, there was a shift in the major FA of MGMG from 16:4 to 16:1 in CC-4533 cells and the proportion of 16:0 was lower, whereas the those of 16:3 and 16:4 was higher in KD-1 and KD-2 MGMG than in CC-4533 MGMG (Figure 6D). The TAG level in KD-1 and KD-2 cells was also slightly lower than that in CC-4533 cells (Supplemental Figure S3). These results are similar to those from cells grown in TAP medium.

For quantitative analysis of MGMG species in total lipids extracted from KD-1, KD-2, and CC-4533 cells, we measured MGMG standard species with the UPLC–ESI–qMS/MS system. We verified the linearity of the standard curve for each C16 MGMG species to evaluate its reliability (Supplemental Figure S4). Next, we quantified C16 MGMG species in total lipids extracted from KD1, KD2, and CC-4533 cells grown in TAP and TAP −P medium (Supplemental Figure S5). For CC-4533, C16 MGMG species accumulated in cells grown in TAP, but their level decreased in the P-depleted cells. KD-2 showed that the amount of C16 MGMG was higher in cells grown in TAP medium than in CC-4533 cells. It was obvious that the amount of 16:4 MGMG was higher in KD-1 and KD-2 MGMG than in CC-4533 MGMG. This result agreed with the GC–FID analysis of lipid composition (Figure 6, B and D). The trends of these changes in FAs suggested that CrLAT1 KD suppressed both MGMG turnover and TAG synthesis by affecting FA flow in Chlamydomonas cells.

## MGMG was decreased in *CrLAT1* overexpression mutants

**Figure 8:**
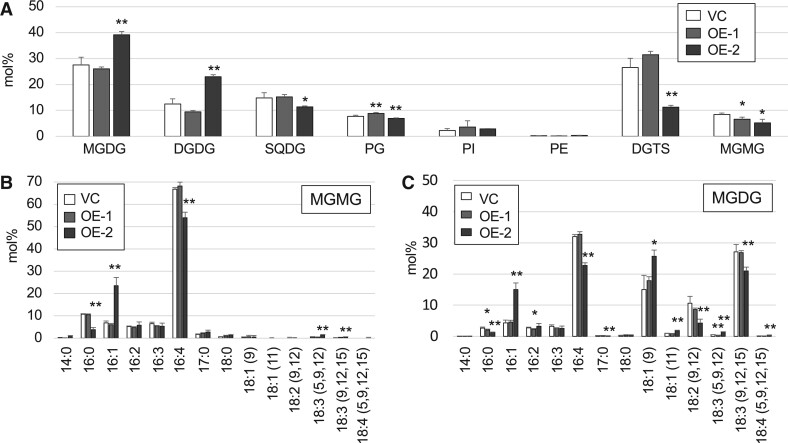
Analyses of major lipid classes and FA composition of MGMG and MGDG in the empty VC and the OE mutants. Cells were cultured in TAP medium for 5 d. Values are the mean ± sd from four independent experiments. Asterisks indicate a statistically significant difference compared with VC based on a two-tailed Student’s *t* test (^*^P < 0.05 and ^**^P < 0.01). A, Relative abundance of the major lipid classes in VC and OE cells. Analyses of the FA composition of the MGMG fraction (B) and MGDG fraction (C) in VC and OE cells.

**Figure 9:**
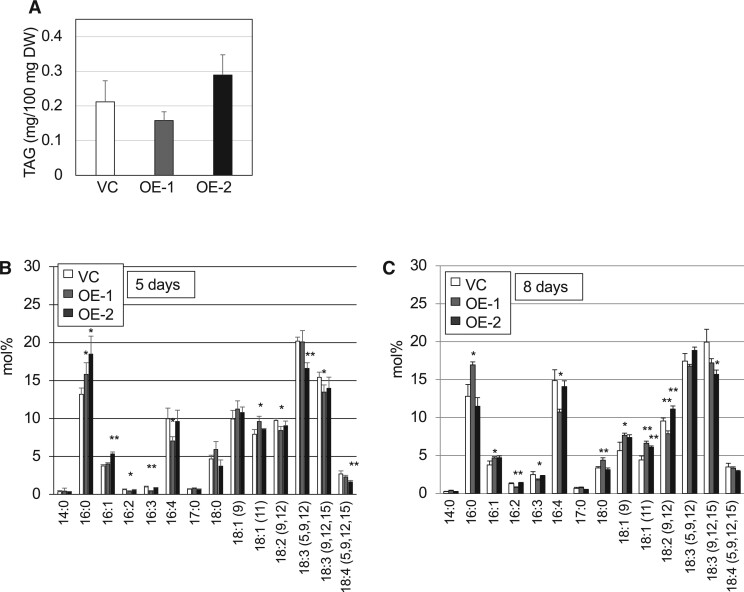
Changes in the TAG content of empty VC and the OE mutant cells. A, Total TAG per DW of cells. B and C, FA composition of the TAG fraction in the VC and the OE mutant cells. Cells were cultured in TAP medium for 5 d (A, B) or 8 d (C). Values are the mean ± sd from four independent experiments. Asterisks indicate a statistically significant difference as compared with the VC based on a two-tailed Student’s *t* test (^*^P < 0.05 and ^**^P < 0.01).

To determine the effects of overexpression (OE) of *CrLAT1* in CC-4533, two OE mutants, OE-1 and OE-2, were produced, and their relative OE of *CrLAT1* after being grown in TAP was measured by RT-qPCR. In OE-1 and OE-2, the *CrLAT1* mRNA level was 10- and 2.5-fold higher, respectively, than that in the vector control (VC; Supplemental Figure S6A). OE-1 and OE-2 cells showed slower growth than the VC cells in TAP −P medium (Supplemental Figure S6B). When cultivated in TAP medium, OE-1 and OE-2 cells were not significantly different in their growth rates relative to that of VC cells. The proportions of MGMG in OE-1 and OE-2 cells grown in TAP was lower than that in VC cells (Figure 8A) in contrast to those in the KD mutants. The proportion of 16:1 was higher, whereas that of 16:4 was lower in OE-2 MGMG relative to VC MGMG (Figure 8B). With respect to MGDG, OE-2 showed changes in its FA composition such that the proportions of 16:1 and 18:1 (9) were higher, whereas that of 18:3 (9, 12, and 15) was lower than that in the VC (Figure 8C;Supplemental Figure S7). We quantified C16 MGMG species in total lipids extracted from OE-1, OE-2, and VC cells grown in TAP and TAP −P medium (Supplemental Figure S8). C16 MGMG species levels accumulated in VC cells grown in TAP but were lower in the P-starved cells. Again, the two lines had a similar tendency such that their C16 MGMG levels were lower relative to the level in VC cells for cells grown in TAP (Supplemental Figure S8, A and B). These results were consistent with the composition of lipids analyzed by GC–FID. Although significant differences in TAG levels were not detected between VC and OE lines (Figure 9A), OE-1 and OE-2 TAG was similar with respect to FA composition in the proportions of polyunsaturated FAs were lower than those in VC TAG at two different time points (Figure 9, B and C); this is in contrast to those in the KD mutants.

The results from the OE lines suggested that *CrLAT1* OE changed the balance between MGMG and MGDG and shifted to enhance the accumulation of de novo-synthesized MGDG. Taken together, these results support our conclusion that CrLAT1 plays an important role in the recycling of MGDG, the major thylakoid membrane, and contributes to the maintenance of lipid homeostasis in *C. reinhardtii*. It is still uncertain whether CrLAT is the lysogalactolipid acyltransferase in *C. reinhardtii.* We have tried to identify the enzyme activity of CrLAT1 but have so far been unsuccessful. We speculate that the substrate *sn*-2 MGMG, which should be used for such assays, may itself have been already desaturated in vivo and thus is easily converted to *sn*-1 MGMG during the preparation step; thus, this makes the activity assay difficult.

## Discussion

In this study, we found a substantial amount of MGMG in the membrane lipids of *C. reinhardtii* and identified a homolog of Arabidopsis LPCAT in *C. reinhardtii* that plays an important role in determining the MGMG level. Both MGDG and TAG are synthesized from FAs produced in the chloroplast, and MGDG may play a crucial role in the supply of FAs for TAG accumulation in response to environmental changes (Li et al., 2012). Indeed, TAG accumulation was decreased in *CrLAT1* KD mutants (Figure 7A;Supplemental Figure S3A).

**Figure 10:**
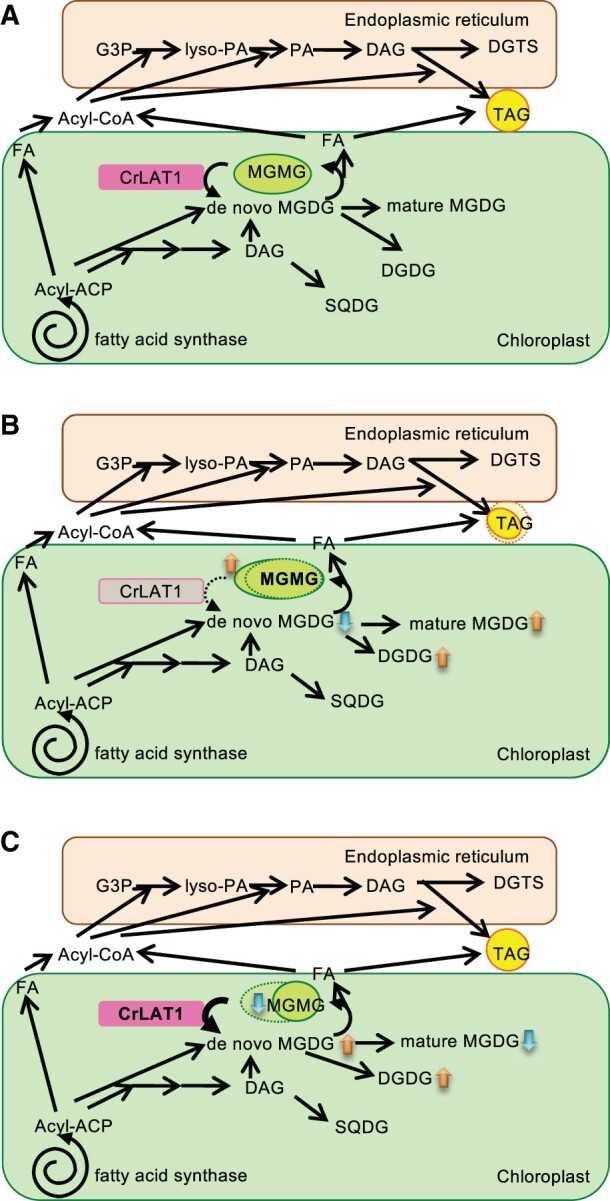
Hypothetical model of the function of CrLAT1 in cellular lipid metabolism in *C. reinhardtii*. A, *Chlamydomonas reinhardtii* CC-4533. CrLAT1 may reacylate MGMG to MGDG to maintain the appropriate level of MGDG in the photosynthetic membrane. B, CrLAT1 KD mutant. MGMG accumulation is increased. TAG accumulation is decreased. C, CrLAT1 OE mutant. MGMG accumulation is decreased. De novo MGDG is enhanced. G3P, glycerol-3-phosphate; PA, phosphatidic acid.

Expression of *CrLAT1* was increased after P depletion but was not increased after N depletion (Figure 4B). This may be because recycling of MGMG to MGDG is one of the acclimation responses to P depletion to maintain appropriate levels of galactolipids in chloroplast membranes, although the gene KD itself does not affect cell growth under these conditions. In fact, *CrLAT1* was coexpressed with *BTA1* and *SQD1* but not with *PGD1* (Supplemental Table S5). In contrast, the expression of *PGD1* was decreased after P depletion (Supplemental Table S2). *PGD1* expression is increased after N deprivation (Miller et al., 2010; Li et al., 2012), which differs from the change in *CrLAT1* expression. Although both CrLAT1 and PGD1 are involved in MGDG/MGMG recycling, these enzymes work in different directions and thus may be regulated differently. Figure 10 provides our model of CrLAT1 function in TAG metabolism. CrLAT1 may reacylate *sn*-2 MGMG to MGDG during normal growth and P depletion to maintain the MGDG of photosynthetic membranes, because *CrMGD1* shows decreased expression based on RNA-seq after P depletion (Hidayati et al., 2019). The MGDG molar ratio is lower and DGTS and SQDG are higher in total lipids after P depletion in *C. reinhardtii* (Hidayati et al., 2019).

PGD1 acts on newly synthesized MGDG containing 18:1/16:0 molecular species, releasing 18:1 from *sn*-1 of the de novo MGDG (Li et al., 2012). Arabidopsis LPCATs catalyze the acylation and de-acylation of both *sn* positions of PC, with a preference for the *sn*-2 position (Lager et al., 2013). Multiple *sn*-2 acyltransferases have been described, whereas there are few reports of *sn*-1 acyltransferases. The activity of an *sn*-1 acyltransferase that targets oxidized lysolipids was reported in platelets (Liu et al., 2019). The activity of a MGMG acyltransferase with a preference for the *sn*-1 position has been described for the cyanobacterium *Anabaena variabilis* ATCC 29211 (Chen et al., 1988). In the case of MGDG FAs, the proportions of 18:1 (9) were lower in the *CrLAT1* KD mutants relative to CC-4533 (Figure 6, C and E;Supplemental Figure S2). Thus, CrLAT1 may have *sn*-1 acyltransferase activity that combines C18:1 (9) and 2-acyl-MGMG, resulting in the production of MGDG that contains 18:1 (9).

It is uncertain why C16:4 was the major component of MGMG. PGD1 hydrolyzes newly synthesized MGDG, which contains fewer unsaturated FAs such as 16:0 and 16:1. It may be possible that accumulated MGMG also could be desaturated to produce more unsaturated C16 FA-containing MGMG. Indeed, OE of *CrLAT1* made the FA composition of MGMG less unsaturated, and, consequently, MGDG also became less unsaturated, suggesting that newly synthesized MGMG has fewer unsaturated FAs such as 16:1.

LPCATs from various land plants may catalyze the reacylation of lysogalactolipids including MGMG. *Dunaliella salina* and *Coccomyxa subellipsoidea*, which have LPCAT1 homologs that are in the same clade as CrLAT1 from *C. reinhardtii* (Figure 4A), contain PC in their membrane lipids (Sheffer et al., 1986; Allen et al., 2017). *Chlorella* sp., which is the other strain of *Chlorella* in Figure 4, also contains PC (White et al., 2019). Although *C. subellipsoidea* contains an SFR2 homolog, there is no evidence that it contains the pathway for converting MGDG to TAG (Allen et al., 2017). There is an intricate relationship between the Lands cycle and the Kennedy pathway of glycerolipid synthesis in developing Arabidopsis seeds (Wang et al., 2012). In Arabidopsis leaves, MGDG production is not impaired in a *lpcat1 lpcat2* double mutant (Wang et al., 2014). In contrast, in the *C. reinhardtii* mutants, KD of *CrLAT1* greatly affected the FA composition of MGDG and suppressed normal growth, indicating that CrLAT1 is important for maintaining the large amount of chloroplast lipid MGDG in Chlamydomonas cells. Our results, together with previous reports for LPCATs in Arabidopsis, also indicate that the acyl recycling mechanism of *C. reinhardtii*, which lacks PC, is different from that of land plants such as Arabidopsis, whereas both recycling mechanisms are important for TAG synthesis in these two organisms. In *Volvox carteri*, which has a homolog that is closely associated with CrLAT1 in *C. reinhardtii* (Figure 4A), PC is not detected in membrane lipids, similar to *C. reinhardtii* (Moseley and Thompson, 1980). The two organisms have almost the same overall genome size, number of protein-coding genes, number of different kinds of protein domains encoded, and distribution of gene family sizes (Prochnik et al., 2010). At a minimum, *Volvox* also may have the ability to recycle MGMG to MGDG by LAT1.

LPCATs have been isolated from the microsomal membranes of developing soybean (*Glycine max*) cotyledons (Rajasekharan and Nachiappan, 1994; Tumaney and Rajasekharan, 1999). In *Portulaca oleracea*, a LPCAT was identified in the microsomal membrane fraction of the leaves and was expressed only in the endoplasmic reticulum (ER; Venkateshwari et al., 2018). In Arabidopsis, a model for the eukaryotic pathway with metabolically distinct pools of PC has been presented, suggesting an underlying spatial organization of PC metabolism as part of the ER–chloroplast metabolic interactions (Karki et al., 2019). It is possible that CrLAT1 is located at chloroplast membranes, if MGMG is reacylated to MGDG by CrLAT1. Although CrLAT1 is predicted to have no chloroplast transit peptide, it may be located in the plastid envelope or at membrane contact sites between the ER and plastid envelope. Cr-PDAT uses chloroplast membrane lipids, particularly MGDG, as substrates to synthesize TAG and may be localized in the chloroplast (Yoon et al., 2012). Based on the electron microscopy examination and the 3D reconstruction by confocal fluorescence microscopy, some lipid droplets are likely to be embedded within chloroplast invaginations in association with the outer envelope of the chloroplast without intervention of ER (Moriyama et al., 2018). Our results are consistent with the traffic from the chloroplast to the lipid droplet without an intervening ER membrane in the previous report. Further research on this unusual type of lysolipid acyltransferase is needed to fully understand the recycling system of the most abundant chloroplast membrane lipid, MGDG.

## Conclusion

Isolated *CrLAT1* KD mutants indicate that CrLAT1 is involved in recycling of MGDG in the chloroplast and maintains lipid homeostasis in *C. reinhardtii*. This finding was not predicted based on knowledge of lipid metabolism in plants containing PC. The proportion of 18:1 (9) MGDG was lower and MGMG was higher in *CrLAT1* KD mutants than in their parental strain, which affected the growth of the mutant cells. In contrast, MGMG was decreased in *CrLAT1* OE mutants. CrLAT1 may have *sn*-1 acyltransferase activity that combines C18:1 (9) and 2-acyl-MGMG, resulting in the production of MGDG that contains 18:1 (9). Further research on CrLAT is needed to fully understand the recycling system of MGDG, the most abundant chloroplast membrane lipid.

## Materials and methods

### Strains and culture conditions

The green alga *C. reinhardtii* strains CC-125 (mt^+^) and CC-408 (mt^−^) were obtained from the *Chlamydomonas* Center (Duke University, Durham NC, USA). *Chlamydomonas reinhardtii* strain CC-4533 (*cw15* mt^−^) and *CrLAT1* KD mutants (KD-1, LMJ.RY0402.059369; KD-2, LMJ.RY0402.165883) were obtained from the Chlamydomonas Genetics Center (https://www.chlamycollection. org). *Chlamydomonas reinhardtii* cells were cultured at 23°C under continuous white light (20–40 μmol photons/m^2^ s^−1^) with shaking. TAP medium (Gorman and Levine, 1965) was used for standard growth. Nutrient deficiency was induced as described in Iwai et al. (2014).

### Chemicals

LC/MS solvents

LC/MS-grade isopropanol and methanol, analytical-grade formic acid, and 25% (v/v) aqueous ammonium hydroxide were obtained from Wako Pure Chemical Industries (Osaka, Japan). Water was purified with a Direct-Q UV3 water purification system (resistivity, 18.2 MΩ cm; Millipore, Bedford, MA, USA).

Standards

To prepare standard lysogalactolipids for LC–MS/MS, total lipid was extracted from *C. reinhardtii* cells, and MGDG was separated by TLC using the solvent system chloroform/methanol/water (65:25:2, v/v/v). The MGDG band was isolated and recovered from the TLC plate (Merck, Germany) by extraction with chloroform/methanol (1:1, v/v). The organic solvent was removed under an N_2_ stream, and then 4 mg MGDG was resuspended in 3.5 mL of 0.1 M phosphate-buffered saline (PBS; pH 7.4) with 0.277% (w/v) Triton X-100 and dispersed by sonication for 6 s × 10 on ice. Then, 1 mL PBS was added, along with 30 mg *R. arrhizus* lipase (∼10 U mg^−1^; Sigma-Aldrich, St. Louis, MO, USA) dissolved in 0.5 mL of PBS with 5 μL of 0.1 M dithiothreitol. The mixture was sonicated again for 5 s and incubated at 22°C for 3 h. The reactions were then stopped by the addition of 5 mL of 0.1 N acetic acid. Then, MGMG was resolved by TLC. The MGMG band was isolated and recovered from silica gel by extraction with chloroform/methanol (1:1, v/v). After the organic solvent was removed under a N_2_ stream, chloroform/methanol (2:1, v/v) was added to yield a final concentration of 10 mg mL^−1^ MGMG. The MGMG species were then quantified by GC–FID (Iwai et al., 2014).

### Lipid analysis

Lipid extraction was done according to Iwai et al. (2014). The lipids were dissolved in chloroform/methanol (2:1, v/v) and stored at −20°C. Lipid solution was spotted on a TLC plate at a position 2 cm by 2 cm from one corner. The first dimension was developed with chloroform/methanol/water (65:25:4, v/v/v). After the plate was dried for 1 h, the second dimension was developed with chloroform/methanol/isopropylamine/28% ammonia solution (65:35:0.5:5, v/v/v/v; Hofmann and Eichenberger, 1996). The separated lipids were visualized under ultraviolet light after spraying the TLC plates with 0.001% (w/v) primuline (Tokyo Chemical Industry Co, Ltd., Tokyo, Japan) in 80% (v/v) acetone. The glycolipids were stained by spraying with anthrone–sulfuric acid (Radin et al., 1955). Lipids were individually scraped off the TLC plates and analyzed with the methods described in Iwai et al. (2014).

### LC–MS conditions

Chromatographic separation was carried out on an ACQUITY UPLC system (Waters, MA, USA) using a UPLC BEH C18 column (2.1 × 100 mm) with a 1.7-μm particle size (Waters). MS was performed with an ACQUITY TQD tandem quadrupole mass spectrometer (Waters). All system controls and data analyses were processed by MassLynx software with QuanLynx version 4.1 (Waters). Chromatographic separation was conducted with isocratic elution using solvent A: 0.028% (v/v) ammonium, 0.2% (v/v) formic acid in isopropanol/methanol/water (50/10/40, v/v/v) at 45°C at a flow rate of 0.1 mL min^−1^. For determination of the concentration of lysogalactolipids in the total lipid sample, 10 μL of the sample was mixed with 1.5 mL solvent A and passed through a membrane filter (FILTSTAR Syringe Filter Hydrophobic PTFE, Starlab Scientific). From the collected sample, 10 μL was injected onto the UPLC/MS/MS system. Each eluate from the UPLC was directly placed into the ESI ion source of the mass spectrometer. The MGMG species were analyzed as the ammonium adducts using ESI conditions in the positive ion mode. The conditions were as follows: spray voltage, 3.5 kV; source and desolvation temperatures, 120°C and 250°C, respectively; cone gas and desolvation gas flows, 50 and 500 L h^−1^, respectively. The extractor voltage was 3 V, and the RF lens voltage was 0.1 V. The collision gas was argon at 0.24 mL min^−1^. Ion energy 1 and 2 were both set at 1.0, and LM 1 and LM 2 resolutions were both set at 14. The MGMG species were checked in Daughter Scan mode (50–540 *m*/*z*) and detected in multiple reaction monitoring (MRM) mode. Mass transitions are shown in Supplemental Table S6. The cone voltage and collision energy were 20 and 10 V, respectively.

### NMR analysis

The UK was dried in vacuo and dissolved in CD_3_OD. ^1^H NMR spectra were measured at 500 MHz on a Varian Unity INOVA 500 spectrometer. The solvent peak in CD_3_OD (*δ* = 3.31 ppm) was used as the standard signal. Chemical shifts are expressed in parts per million with reference to the standard signal. NMR signals for the UK were as follows: ^1^H NMR (500 MHz, CD_3_OD) δ 5.43–5.27 (m, 8H), 4.23 (d, *J* = 7.6 Hz, 1H, H-1’), 4.16 (m, 2H, H-1), 4.03–3.97 (m, 1H, H-2), 3.92 (dd, *J* = 10.4 Hz, *J* = 5.1 Hz, 1H, H-3), 3.82 (d, *J* = 3.3 Hz, 1H, H-4’), 3.79–3.70 (m, 2H, H-6’), 3.65 (dd, *J* = 10.5 Hz, *J* = 4.6 Hz, 1H, H-3), 3.53 (m, 2H, H-2’, H-5’), 3.47 (dd, *J* = 9.7 Hz, *J* = 3.4 Hz, 1H, H-3’), 2.91–2.77 (m, 6H), 2.41 (m, 4H), 2.13–2.05 (m, 2H), 0.98 (t, *J* = 7.5 Hz, 3H). The signals were identical to those reported previously (Rho et al., 1997), although we note that all chemical shift values are shifted ca. –0.03 ppm as compared with those reported previously.

### Phylogenetic analyses and bioinformatic analyses

Amino acid sequences of putative LPCAT homologs from algae and plants were aligned using the online version of MAFFT (Katoh et al., 2019; https://mafft.cbrc.jp/alignment/server/). Regions of low sequence conservation were eliminated with the Gblocks server version 0.91b (Talavera and Castresana, 2007; http://molevol.cmima.csic.es/castresana/Gblocks_server.html). Phylogenetic analyses were performed using the maximum likelihood method in MEGA X (Kumar et al., 2018; Stecher et al., 2020). Evolutionary history was inferred using the maximum likelihood method and JTT matrix-based model (Jones et al., 1992) with 1,000 bootstraps.

ChloroP version 1.1 (http://www.cbs.dtu.dk/services/ChloroP/), PredAlgo (Tardif et al., 2012; https://giavapIgenomes.ibpc.fr/cgiIbin/predalgodb.perl?page=main) and TargetP version 2 (http://www.cbs.dtu.dk/services/TargetP-2.0/index.php) were used in bioinformatic analyses of protein subcellular localization.

### Co-expression analysis

Coexpression data used in this study were downloaded from ALCOdb (http://alcodb.jp, Cre-R1-15-08; Aoki et al., 2016) and ATTED-II (http://atted.jp, version 9.2; Obayashi et al., 2018).

### Mutant isolation

Genomic DNA was isolated by Tris–EDTA (pH 8.0)—saturated phenol/chloroform extraction. For genotyping, the primer sets shown in Supplemental Table S7 were used.

### Overexpression

To create cDNA clones for CrLAT1 OE, RNA was prepared by the phenol/chloroform method from *C. reinhardtii* strain CC-408. Reverse transcription was conducted with Superscript II reverse transcriptase (Invitrogen, Waltham, MA, USA) to obtain cDNA. *CrLAT1* was amplified by PCR from cDNA using PrimeSTAR GXL DNA Polymerase (Takara, Shiga, Japan) and the primer sets containing *Pst*I and *Eco*RI restriction sites are shown in Supplemental Table S7. The PCR products were cloned into pZErO-2 (Invitrogen). To construct the OE plasmid, *Pst*I and *Eco*RI restriction sites were replaced with the truncated miRNA precursor cre-MIR1157 in pChlamiRNA3 (Molnar et al., 2009). After DNA sequencing, the PCR products and the modified pChlamiRNA3 vector were digested with restriction endonucleases *Pst*I and *Eco*RI and ligated using Mighty Mix (Takara). Additional DNA sequencing was carried out to confirm the vector construct, which was then transformed into *C. reinhardtii* strain CC-4533 by electroporation as described (Iwai et al., 2014). The modified pChlamiRNA3 vector, which resulted in removal of the truncated miRNA precursor cre-MIR1157, was used as the VC. Transformants were selected on TAP plates with paromomycin (10 μg mL^−1^).

### Analysis of gene expression

Total RNA was extracted and quantified as described (Iwai et al., 2014). *CrLAT1* expression was normalized to G protein beta subunit-like polypeptide (*CBLP*) mRNA expression. Primers are listed in Supplemental Table S7.

### Statistical analyses

Graphs represent the means ± sd of four biological-independent experiments. The statistical significance was assayed using means a two-tailed Student’s *t* test.

### Accession numbers

Sequence data from this article can be found in the Phytozome version 12 (http://phytozome.jgi.doe.gov/pz/portal.html) under accession numbers: Cre03.g193500 (*PGD1*), Cre06.g278222 (*CBLP*), Cre07.g324200 (*BTA1*), Cre12.g537641 (*LAT1*), Cre13.g585301 (*MGD1*), and Cre16.g656400 (*SQD1*).

## Supplemental data

The following materials are available in the online version of this article.

**Supplemental Figure S1**. Fractionation by 2D-TLC of total lipids from cells of *C. reinhardtii* CC-125 grown in TAP medium.

**Supplemental Figure S2**. Analysis of FA composition of MGDG from *C. reinhardtii* CC-4533 and *CrLAT1* KD mutant cells.

**Supplemental Figure S3**. Changes in the TAG content of *C. reinhardtii* CC-4533 and the *CrLAT1* KD mutant cells.

**Supplemental Figure S4**. Standard curves for each C16 MGMG species.

**Supplemental Figure S5**. MGMG species in total lipids extracted from *C. reinhardtii* CC-4533 and the *CrLAT1* KD cells grown in TAP and TAP −P (−P) medium for different time periods.

**Supplemental Figure S6**. RT-qPCR showing the induction of CrLAT1 expression (A) and growth (B) of VC and OE mutants.

**Supplemental Figure S7**. Analysis of FA composition of MGDG in empty VC cells and the OE mutant cells.

**Supplemental Figure S8**. MGMG species in total lipids extracted from empty VC cells and the OE mutant cells grown in TAP and TAP −P (−P) medium.

**Supplemental Table S1**. Prediction results of CrLAT1 subcellular localization according to various algorithms.

**Supplemental Table S2**. Normalized read count for MGD1, PGD1, and LAT1.

**Supplemental Table S3**. At1g12640 coexpressed gene list.

**Supplemental Table S4**. At1g63050 coexpressed gene list.

**Supplemental Table S5**. Cre12g537641 coexpressed gene list.

**Supplemental Table S6**. The mass of different MGMG species monitored in MRM mode.

**Supplemental Table S7**. Primer sequences used in this study.

## Supplementary Material

kiab340_Supplementary_Data

## Notes

M.I. and H.O. designed this work; M.I., Y.Y.O., K.A., and T.K. performed the experiments; M.I., Y.Y.O., T.K., H.Y., and H.O. analyzed the data; M.I. wrote the manuscript with contributions from Y.Y.O., K.A., T.K., H.Y., M.S., and H.O.

The author responsible for distribution of materials integral to the findings presented in this article in accordance with the policy described in the Instructions for Authors (https://academic.oup.com/plphys/pages/general-instructions) is Hiroyuki Ohta (ohta.h.ab@m.titech.ac.jp).
