# Supplementary material for: Recycling of the major thylakoid lipid MGDG and its role in lipid homeostasis in Chlamydomonas reinhardtii
Source: Plant Physiol. 2021 Jul 23;187(3):1341–56. doi: 10.1093/plphys/kiab340 (PMC8566231; doi:10.1093/plphys/kiab340)
Supplement: kiab340_Supplementary_Data [file kiab340_supplementary_data.pdf]

1. free FA
2. MGDG
3. DGTS
4. DGDG
5. PI
6. SQDG
7. PG
8. PE

A, unknown

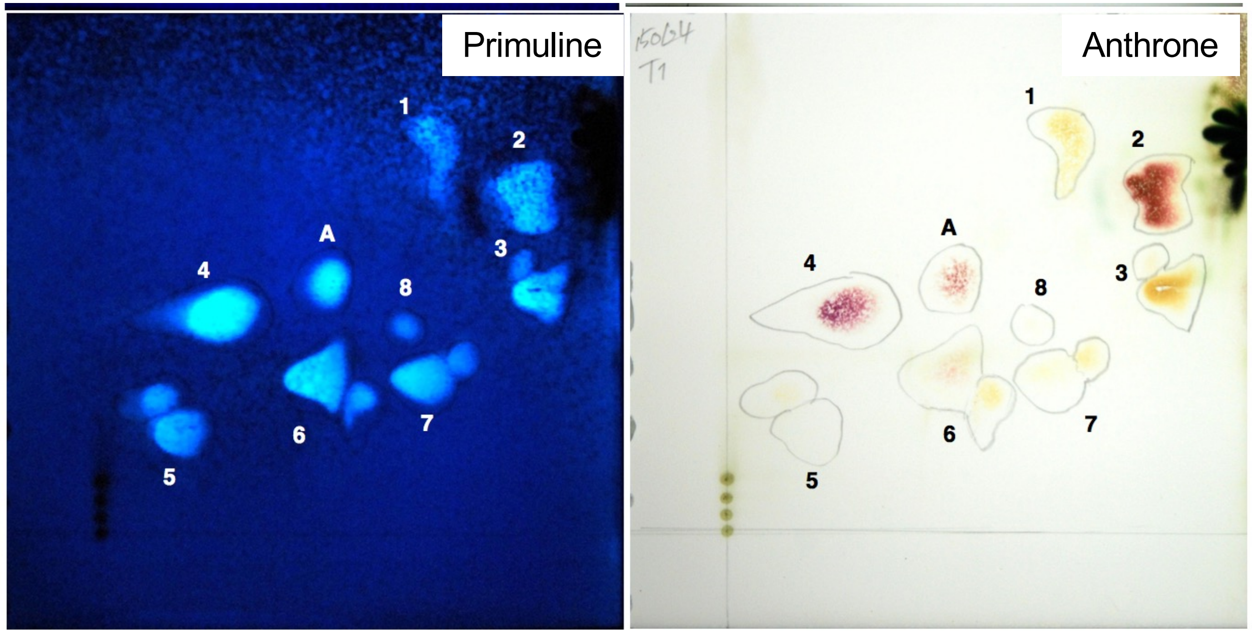

Supplemental Figure S1 Fractionation by 2D-TLC of total lipids from cells of *C. reinhardtii* CC-125 grown in TAP medium.
Chromatography was performed in chloroform/methanol/water (65:25:4, v/v/v) for the first dimension and chloroform/methanol/isopropylamine/ammonia water (65:35:0.5:5, v/v/v/v) for the second dimension. Primuline was used to stain all lipids, and galactolipids were stained with anthrone–sulfuric acid reagent. DGDG, digalactosyldiacylglycerol; DGTS, diacylglyceryltrimethylhomoserine; free FA, free fatty acids; MGDG, monogalactosyldiacylglycerol; PE, phosphatidylethanolamine; PG, phosphatidylglycerol; PI, phosphatidylinositol; SQDG, sulfoquinovosyldiacylglycerol; unknown, unknown lipid.

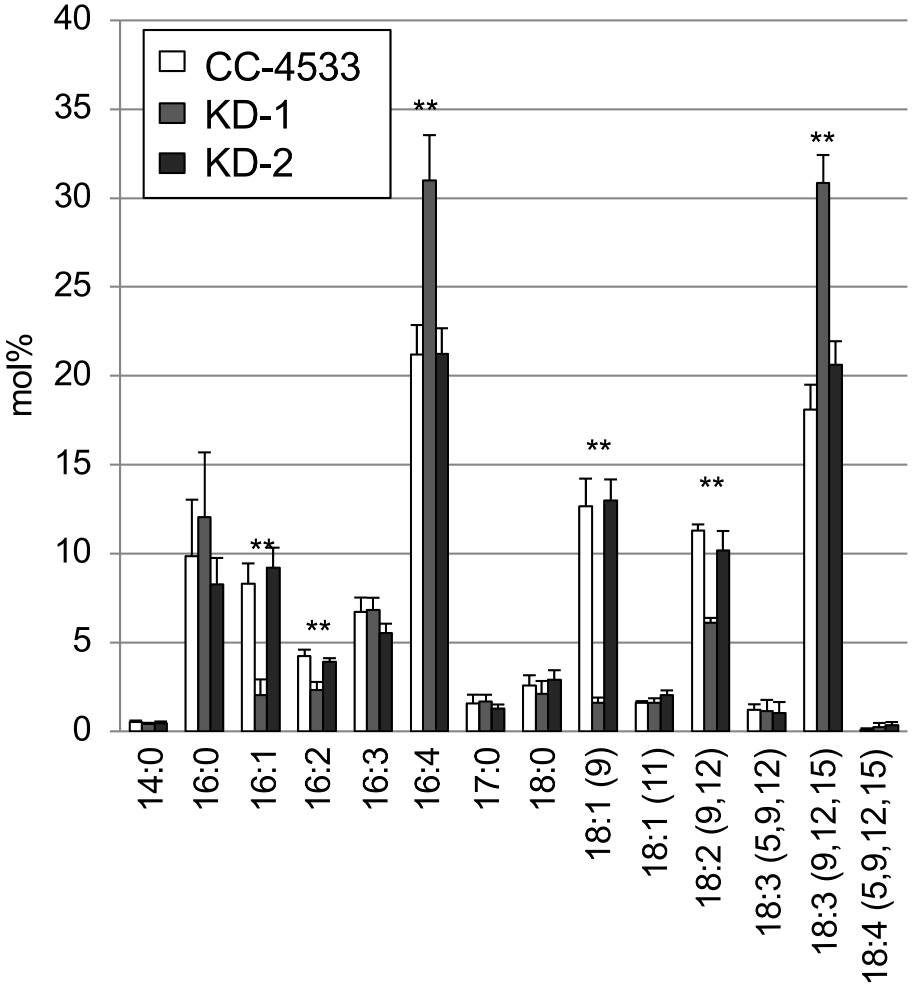

Supplemental Figure S2 Analysis of fatty acid composition of MGDG from *C. reinhardtii* CC-4533 and *CrLAT1* knockdown (KD) mutant cells.

Fatty acid composition of the MGDG fraction. Cells were cultured in TAP medium for 8 days. Values are the mean ± SD from four independent experiments. Asterisks indicate a statistically significant difference as compared with CC-4533 based on a two-tailed Student's t-test (*P < 0.05 and **P < 0.01).

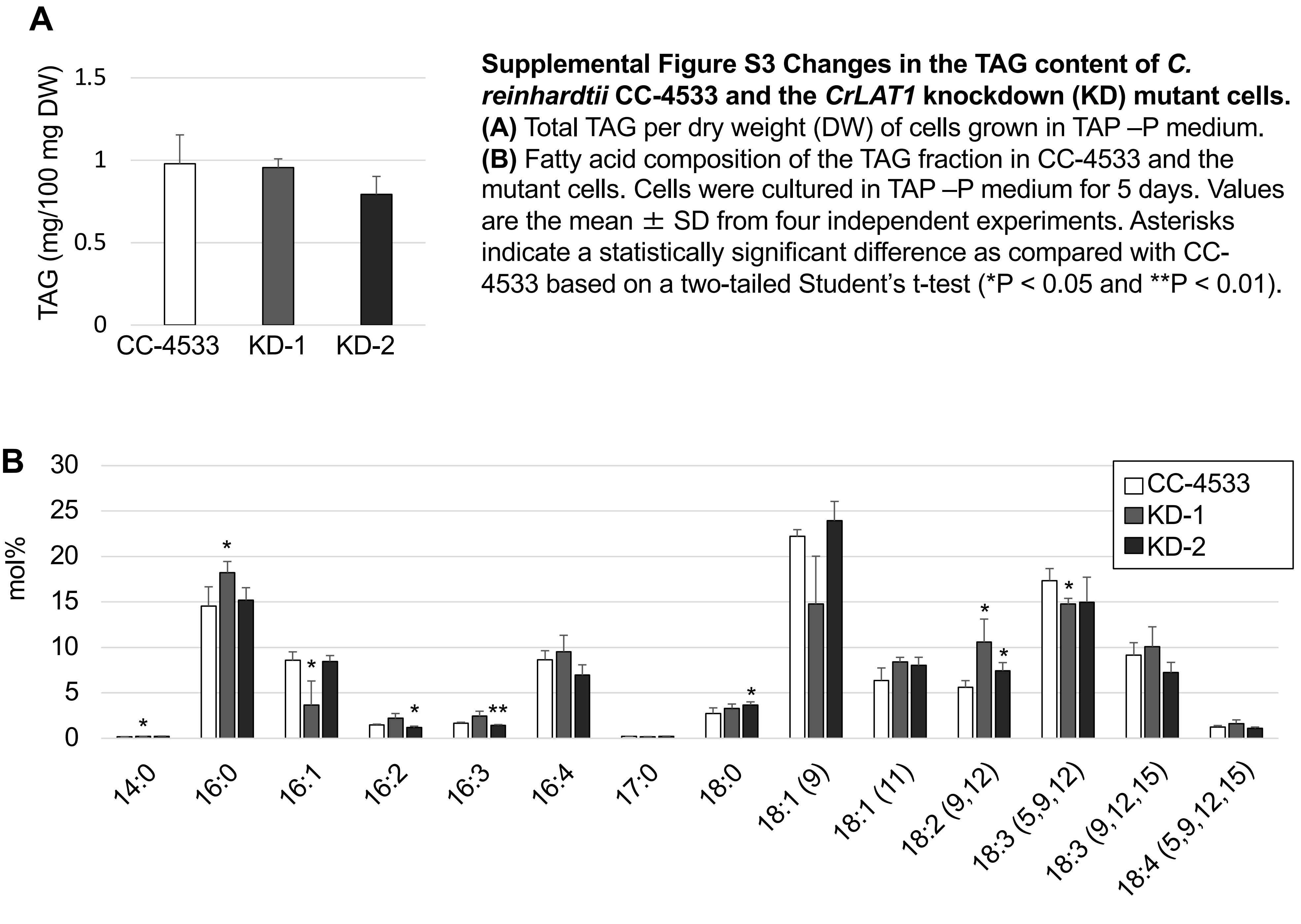

Supplemental Figure S3 Changes in the TAG content of *C. reinhardtii* CC-4533 and the *CrLAT1* knockdown (KD) mutant cells. **(A)** Total TAG per dry weight (DW) of cells grown in TAP –P medium. **(B)** Fatty acid composition of the TAG fraction in CC-4533 and the mutant cells. Cells were cultured in TAP –P medium for 5 days. Values are the mean ± SD from four independent experiments. Asterisks indicate a statistically significant difference as compared with CC-4533 based on a two-tailed Student's t-test (*P < 0.05 and **P < 0.01).

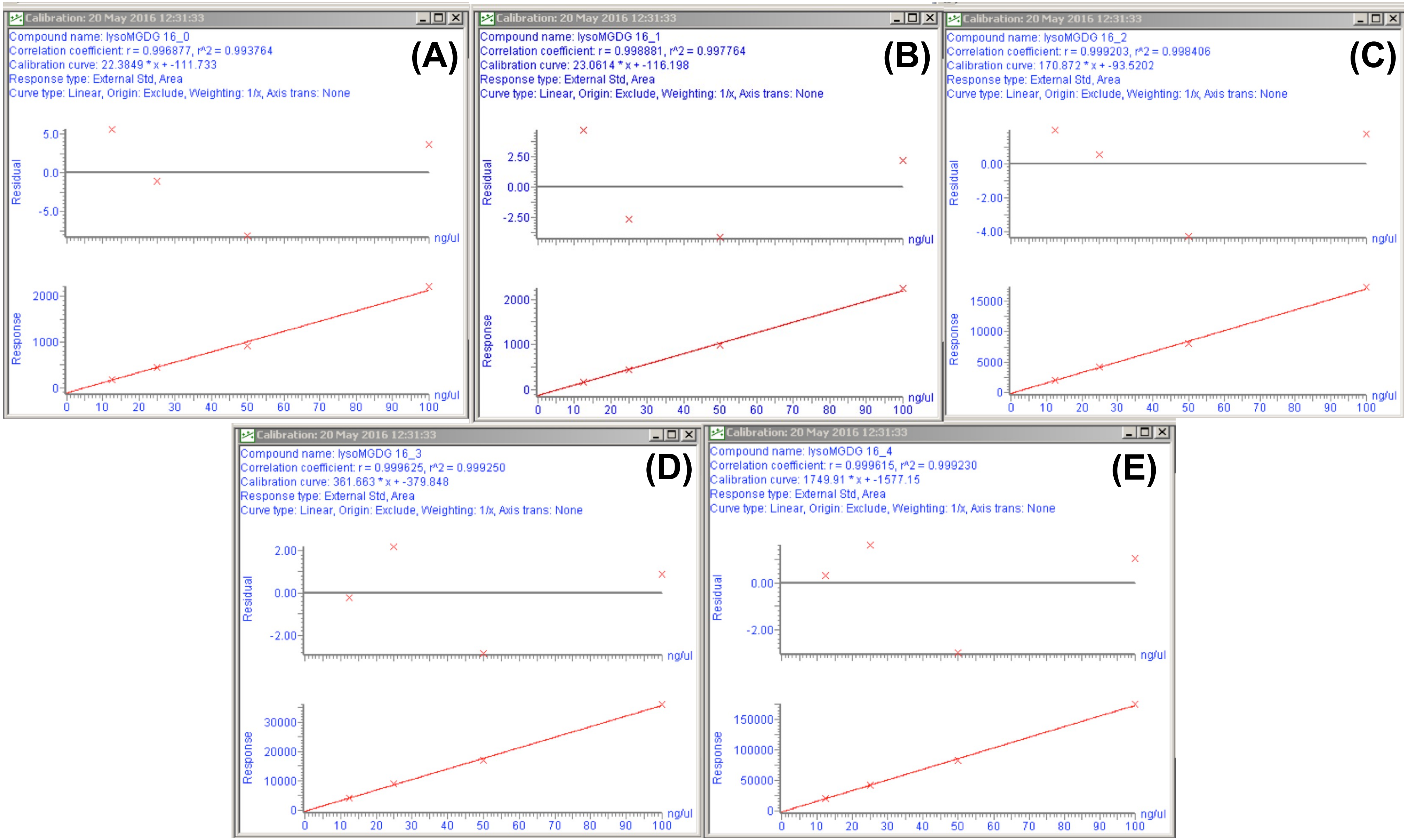

Supplemental Figure S4 Standard curves for each C16 MGMG species.

Standard curves were processed by MassLynx software with QuanLynx (version 4.1). **(A)** C16:0 MGMG **(B)** C16:1 MGMG **(C)** C16:2 MGMG **(D)** C16:3 MGMG **(E)** C16:4 MGMG. Each standard curve was linear for an injected volume of 10 µl with $r^2 > 0.99$.

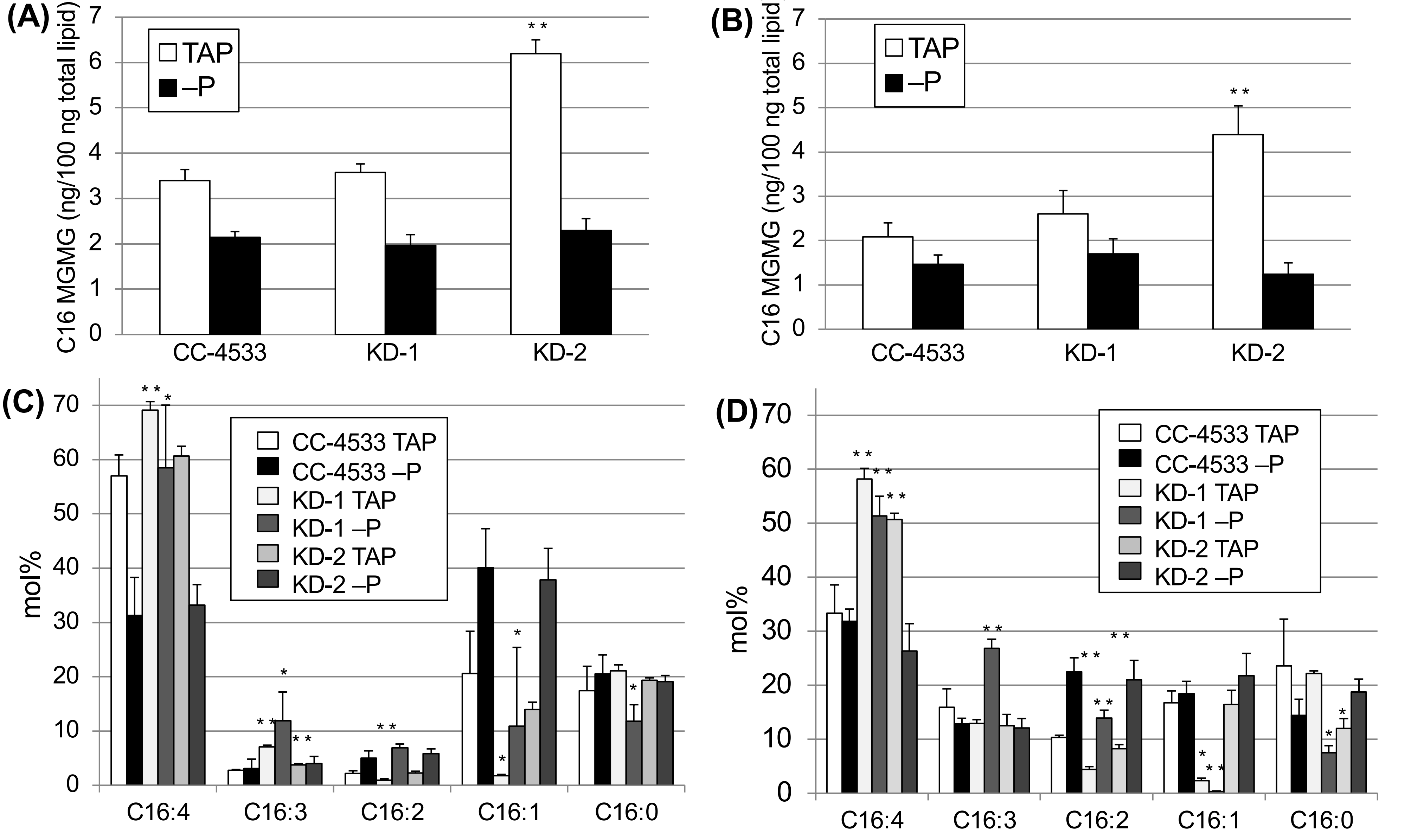

Supplemental Figure S5 MGMG species in total lipids extracted from *C. reinhardtii* CC-4533 and the *CrLAT1* knockdown (KD) cells grown in TAP and TAP –P (–P) medium for different time periods.

(A, B) MGMG per total lipid. **(C, D)** Fatty acid composition of MGMG in total lipid. Cells were cultured for 5 days **(A, C)** or 8 days **(B, D)**. Values are the mean ± SD from four independent experiments. Asterisks indicate a statistically significant difference as compared with CC-4533 based on a two-tailed Student's t-test (*P < 0.05 and **P < 0.01).

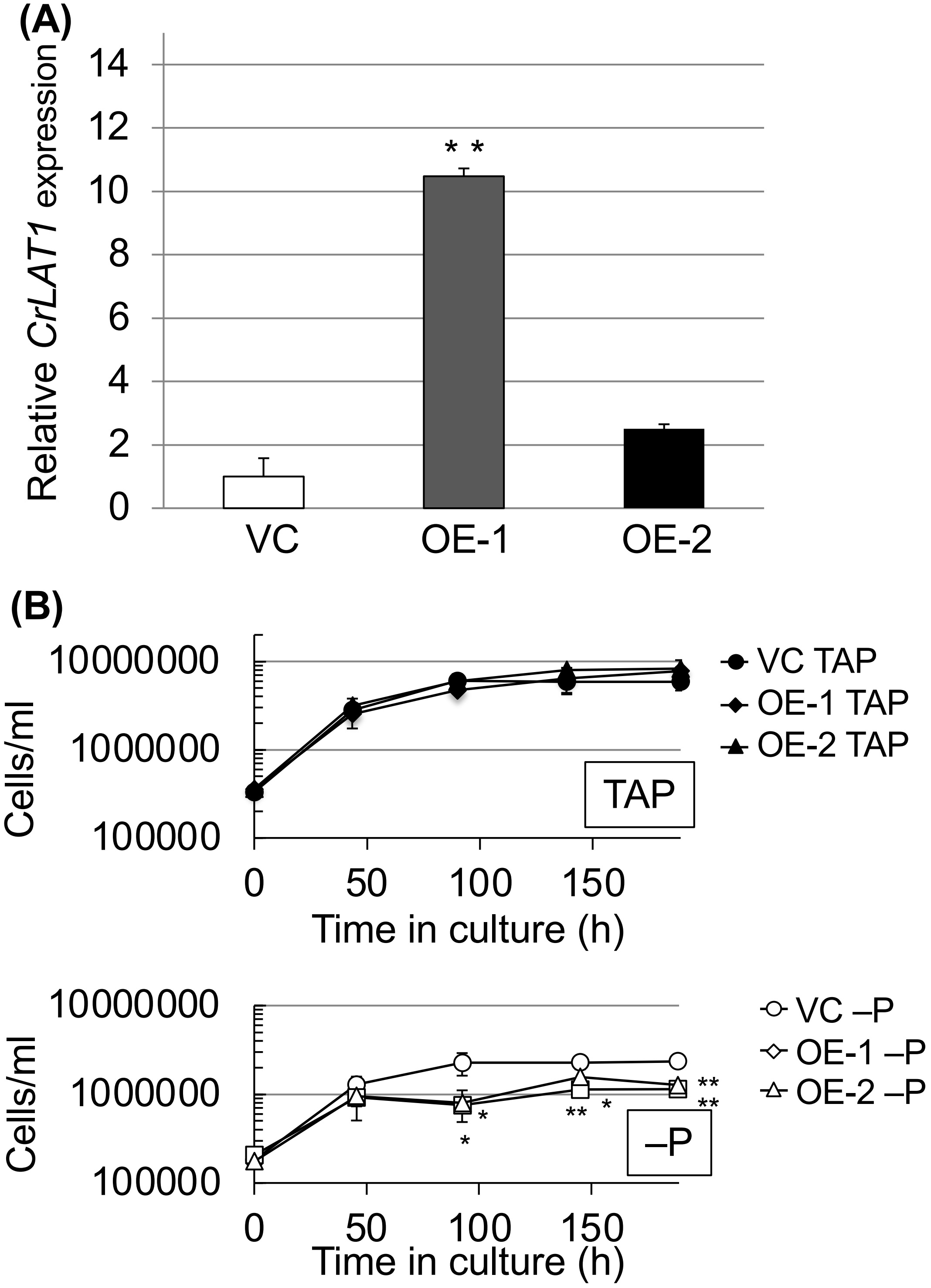

Supplemental Figure S6 Reverse transcription quantitative PCR showing the induction of *CrLAT1* expression (A) and growth (B) of VC and OE mutants. **(A)** The relative levels of *CrLAT1* mRNA in the overexpression (OE) cell lines were assessed under standard growth conditions. Cells were cultured for 5 days in TAP medium. The values were normalized to the expression of *CBLP*. Error bars represent standard errors based on four biological replicates. Asterisk indicate a statistically significant difference as compared with the VC (vector control) based on a two-tailed Student's t-test (**P < 0.01). **(B)** Growth of VC and OE mutants grown in TAP medium or under P starvation. *C. reinhardtii* cells precultured to logarithmic phase in TAP medium were then inoculated into TAP or TAP –P (–P) medium. Values represent the mean $\pm$ SD from four independent replicates. Asterisks indicate a statistically significant difference compared with VC based on a two-tailed Student's t-test (*P < 0.05 and **P < 0.01).

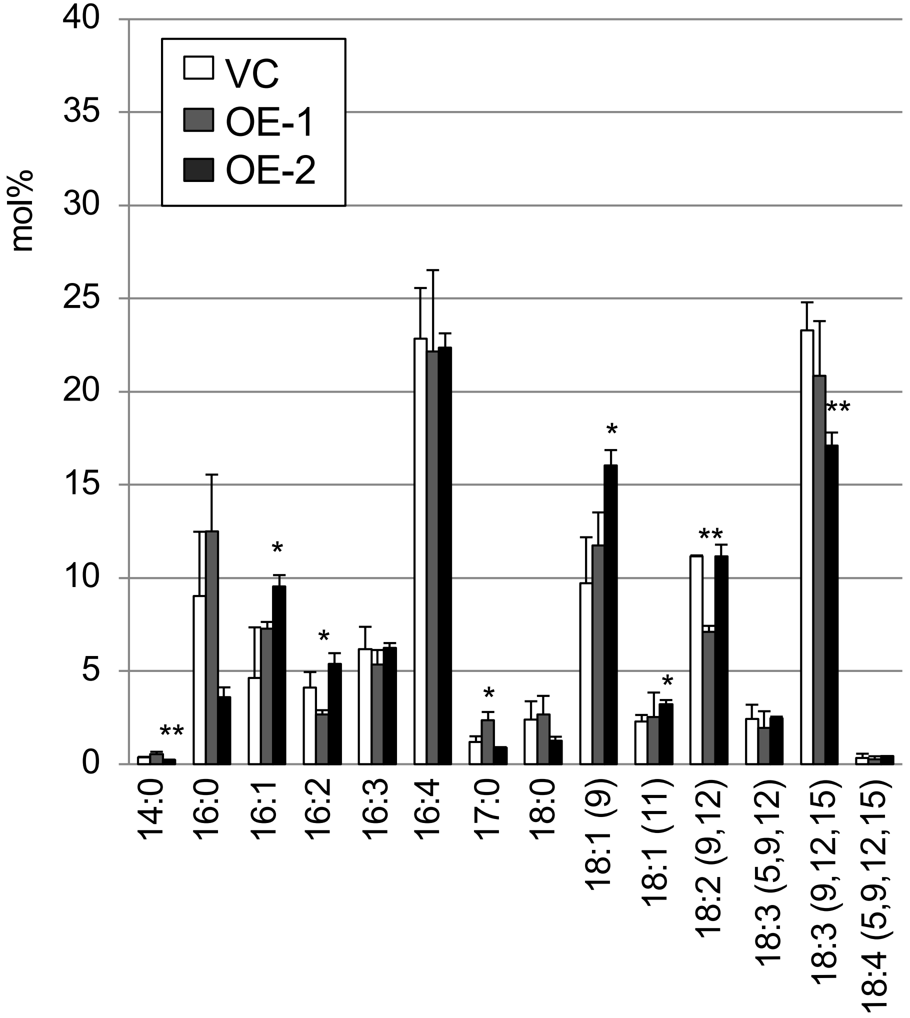

Supplemental Figure S7 Analysis of fatty acid composition of MGDG in empty vector control (VC) and the overexpression (OE) mutant cells.

(A) Fatty acid composition of the MGDG fraction. Cells were cultured in TAP medium for 8 days. Values are the mean ± SD from four independent experiments. Asterisks indicate a statistically significant difference as compared with VC cells based on a two-tailed Student’s t-test (*P < 0.05 and **P < 0.01).

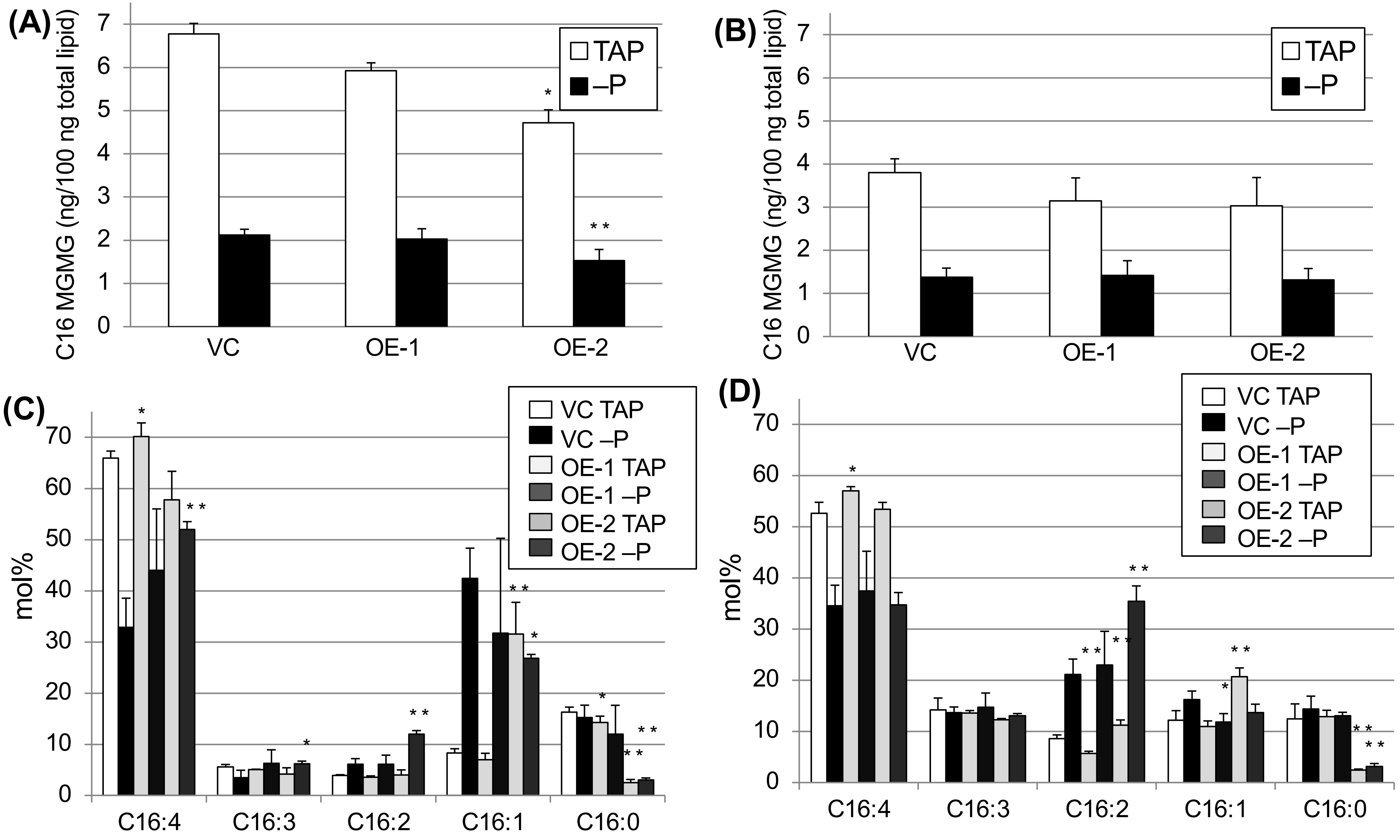

Supplemental Figure S8 MGMG species in total lipids extracted from empty vector control (VC) and the overexpression (OE) mutant cells grown in TAP and TAP –P (–P) medium.

(A, B) MGMG per total lipid. **(C, D)** Fatty acid composition of MGMG in total lipid. Cells were cultured for 5 days **(A, C)** or 8 days **(B, D)**. Values are the mean ± SD from four independent experiments. Asterisks indicate a statistically significant difference as compared with the VC based on a two-tailed Student's t-test (*P < 0.05 and **P < 0.01).

Supplemental Table S1 Prediction results of CrLAT1 subcellular localization according to various algorithms.

	PredAlgo				TargetP 2.0					ChloroP 1.1	
	Mscore	Cscore	Spscore	Target	Other	Signal peptide	Mitochondrial transfer peptide	Chloroplast transfer peptide	Thylakoid luminal transfer peptide	Score	cTP
CrLAT1	0.1900826	0.0366751	0.3914019	SP	0.9985	0.0007	0.0004	0.0003	0	0.432	-

PredAlgo

M; mitochondrion

C; chloroplast

SP; secretory pathway

ChloroP 1.1

cTP; chloroplast transit peptides

Score is the output score from the second step network. The prediction cTP/no cTP is based solely on this score.

cTP tells whether or not this is predicted as a cTP-containing sequence; "-" means that is predicted not to contain a cTP.

Supplemental Table S2 Normalized read count for MGD1, PGD1 and LAT1.

			Normalized read count											
Gene ID	*A. thaliana annotation*	Gene symbol	C9 4 d, +P	C9 4 d, –P	CC-125 5 d, +P	CC-125 5 d, –P	CW15 8 d, +P	CC-4334 8 d, +P	CW15 13 d, +P	CC-4334 13 d, +P	CW15 8 d, –P	CC-4334 8 d, –P	CW15 13 d, –P	CC-4334 13 d, –P
Cre13.g585301	monogalactosyl diacylglycerol synthase 1	MGD1*	1282	540	871	462	2980	1698	791	1831	689	745	702	713
Cre03.g193500	alpha/beta-hydrolases superfamily protein	PGD1	2508	391	1091	815	2539	1389	967	4414	408	976	163	811
Cre12.g537641	MBOAT family protein	LAT1	221	905	226	619	543	1083	494	480	1651	747	1168	791

MBOAT, membrane-bound *O*-acyl transferase

C9, CC-125, CW15, and CC-4334, *C. reinhardtii* strains

Cells were cultured for 4, 5, 8, and 13 d under TAP (+P) and TAP –P (–P) conditions.

*Nur et al. 2019

Supplemental Table S3 At1g12640 Coexpressed Gene List.

Rank	Locus	Alias	Function
0	At1g12640	LPLAT1	MBOAT (membrane bound O-acyl transferase) family protein
1	At5g54870		
2	At3g03720	CAT4	cationic amino acid transporter 4
3	At5g11960	DUF803	Protein of unknown function (DUF803)
4	At3g17810	PYD1	pyrimidine 1
5	At5g45410		
6	At3g09320	zinc finger	DHHC-type zinc finger family protein
7	At2g02040	PTR2-B	peptide transporter 2
8	At3g51730	saposin B	saposin B domain-containing protein
9	At3g02740	protease	Eukaryotic aspartyl protease family protein
10	At3g63310	BIL4	Bax inhibitor-1 family protein
11	At3g04090	SIP1A	small and basic intrinsic protein 1A
12	At4g19640	RABF2B	Ras-related small GTP-binding family protein
13	At1g04970	glycoprotein	lipid-binding serum glycoprotein family protein
14	At3g19950	RING/U-box	RING/U-box superfamily protein
15	At3g51130		
16	At1g54710	G18H	homolog of yeast autophagy 18 (ATG18) H
17	At5g01800	saposin B	saposin B domain-containing protein
18	At1g23440	I-like	Peptidase C15, pyroglutamyl peptidase I-like
19	At5g51710	KEA5	K+ efflux antiporter 5
20	At3g58750	CSY2	citrate synthase 2
21	At3g25585	AAPT2	aminoalcoholphosphotransferase
22	At1g32790	CID11	CTC-interacting domain 11
23	At1g02816	DUF538	Protein of unknown function, DUF538
24	At5g07220	BAG3	BCL-2-associated athanogene 3
25	At5g02040	PRA1.A1	prenylated RAB acceptor 1.A1
26	At3g61070	PEX11E	peroxin 11E
27	At4g28770	Tetraspanin	Tetraspanin family protein
28	At3g26100	RCC1	Regulator of chromosome condensation (RCC1) family protein
29	At2g02870	Galactose oxidase/kelch repeat	Galactose oxidase/kelch repeat superfamily protein
30	At3g30390	transporter	Transmembrane amino acid transporter family protein
31	At1g16180	Serinc-domain containing serine and sphingolipid biosynthesis	Serinc-domain containing serine and sphingolipid biosynthesis protein
32	At4g39140	RING/U-box	RING/U-box superfamily protein
33	At1g77770	DUF1644	Protein of unknown function (DUF1644)
34	At3g56310	Melibiase	Melibiase family protein
35	At4g16110	RR2	response regulator 2
36	At5g59840	GTP-binding	Ras-related small GTP-binding family protein
37	At2g31350	GLX2-5	glyoxalase 2-5
38	At1g68000	PIS1	phosphatidylinositol synthase 1
39	At2g39780	RNS2	ribonuclease 2
40	At5g27150	NHX1	Na+/H+ exchanger 1
41	At1g71190	SAG18	senescence associated gene 18
42	At2g43790	MPK6	MAP kinase 6
43	At3g17000	UBC32	ubiquitin-conjugating enzyme 32
44	At1g47128	RD21A	Granulin repeat cysteine protease family protein
45	At5g49890	CLC-C	chloride channel C
46	At4g32680		
47	At1g29330	ERD2	ER lumen protein retaining receptor family protein
48	At2g28110	IRX7	Exostosin family protein
49	At4g21160	ZAC	Calcium-dependent ARF-type GTPase activating protein family
50	At5g64930	HYS1	CPR5 protein, putative
51	At1g23040	glycoprotein	hydroxyproline-rich glycoprotein family protein
52	At5g42090	Lung seven transmembrane receptor	Lung seven transmembrane receptor family protein
53	At4g35860	GB2	GTP-binding 2
54	At5g54080	HGO	homogentisate 1,2-dioxygenase
55	At1g02305	Cysteineases	Cysteine proteinases superfamily protein
56	At4g14950	KMS1	SNARE associated Golgi protein family
57	At2g01540	Calcium-dependent lipid-binding	Calcium-dependent lipid-binding (CaLB domain) family protein
58	At4g16760	ACX1	acyl-CoA oxidase 1
59	At1g25520	UPF0016	Uncharacterized protein family (UPF0016)
60	At5g44790	RAN1	copper-exporting ATPase / responsive-to-antagonist 1 / copper-transporting ATPase (RAN1)
61	At3g43230	zinc finger	RING/FYVE/PHD-type zinc finger family protein
62	At1g74020	SS2	strictosidine synthase 2
63	At2g41490	GPT	UDP-glcnac-adolichol phosphate glcnac-1-p-transferase
64	At4g21980	G8A	Ubiquitin-like superfamily protein
65	At3g11900	ANT1	aromatic and neutral transporter 1
66	At3g22290	transporter	Endoplasmic reticulum vesicle transporter protein
67	At4g13270	LEA	Late embryogenesis abundant (LEA) hydroxyproline-rich glycoprotein family
68	At1g75760	ER lumen retaining receptor	ER lumen protein retaining receptor family protein
69	At5g45900	ATG7	ThiF family protein
70	At5g35460		
71	At1g29760	Seipin	Putative adipose-regulatory protein (Seipin)
72	At2g17500	Auxin efflux carrier	Auxin efflux carrier family protein
73	At5g61640	PMSR1	peptidemethionine sulfoxide reductase 1
74	At5g64370	PYD3	beta-ureidopropionase
75	At3g23560	ALF5	MATE efflux family protein
76	At4g31130	DUF1218	Protein of unknown function (DUF1218)
77	At2g23310	RER1C1	Rer1 family protein
78	At1g53580	GLY3	glyoxalase II 3
79	At5g24870	RING/U-box	RING/U-box superfamily protein
80	At3g54680	proteophosphoglycan	proteophosphoglycan-related
81	At3g49210	WSD1-like	O-acyltransferase (WSD1-like) family protein
82	At3g53780	RBL4	RHOMBOID-like protein 4
83	At3g51990	kinase	Protein kinase superfamily protein
84	At5g58730	Mik	pfkB-like carbohydrate kinase family protein
85	At2g28370	UPF0497	Uncharacterised protein family (UPF0497)
86	At4g27320	PHOS34	Adenine nucleotide alpha hydrolases-like superfamily protein
87	At2g39970	PXN	Mitochondrial substrate carrier family protein
88	At4g27780	ACBP2	acyl-CoA binding protein 2
89	At1g28960	NUDX15	nudix hydrolase homolog 15
90	At4g40050	DUF3550/UPF0682	Protein of unknown function (DUF3550/UPF0682)
91	At5g58060	YKT61	SNARE-like superfamily protein
92	At5g40470	RNI-like	RNI-like superfamily protein
93	At2g42790	CSY3	citrate synthase 3
94	At5g57740	XBAT32	XB3 ortholog 2 in Arabidopsis thaliana
95	At1g29970	RPL18AA	60S ribosomal protein L18A-1
96	At4g17530	RAB1C	RAB GTPase homolog 1C
97	At5g45550	MOB1-like	Mob1/phocein family protein
98	At1g60430	ARPC3	actin-related protein C3
99	At4g11220	RTNLB2	VIRB2-interacting protein 2
100	At5g18900	2OG	2-oxoglutarate (2OG) and Fe(II)-dependent oxygenase superfamily protein

Supplemental Table S4 At1g63050 Coexpressed Gene List.

Rank	Locus	Alias	Function
0	At1g63050	LPLAT2	MBOAT (membrane bound O-acyl transferase) family protein
1	At4g27080	PDIL5-4	PDI-like 5-4
2	At5g52180		
3	At3g11250	L10	Ribosomal protein L10 family protein
4	At1g18340	transcription	basal transcription factor complex subunit-related
5	At1g48630	RACK1B_AT	receptor for activated C kinase 1B
6	At3g18130	RACK1C_AT	receptor for activated C kinase 1C
7	At1g20950	Phosphofructokinase	Phosphofructokinase family protein
8	At3g28720		
9	At3g49010	RSU2	breast basic conserved 1
10	At3g04840	S3Ae	Ribosomal protein S3Ae
11	At2g01250	L30/L7	Ribosomal protein L30/L7 family protein
12	At2g24170	Endomembrane 70	Endomembrane protein 70 protein family
13	At5g61970	SRP	signal recognition particle-related / SRP-related
14	At4g31700	RPS6A	ribosomal protein S6
15	At1g10030	ERG28	homolog of yeast ergosterol28
16	At5g58420	RPS4A	Ribosomal protein S4 (RPS4A) family protein
17	At1g64650	Major facilitator	Major facilitator superfamily protein
18	At3g52940	HYD2	Ergosterol biosynthesis ERG4/ERG24 family
19	At5g59850	Ribosomal S8	Ribosomal protein S8 family protein
20	At5g46570	BSK2	BR-signaling kinase 2
21	At3g09630	L4/L1	Ribosomal protein L4/L1 family
22	At1g76400	Ribophorin I	Ribophorin I
23	At5g38460	transferase	ALG6, ALG8 glycosyltransferase family
24	At3g01380	hydrolase	transferases;sulfuric ester hydrolases;catalytics;transferases
25	At5g22440	L1p/L10e	Ribosomal protein L1p/L10e family
26	At1g05210	Transmembrane 97	Transmembrane protein 97, predicted
27	At3g62360	Carbohydrate-binding-like fold	Carbohydrate-binding-like fold
28	At5g45600	YAF9a	YEATS family protein
29	At1g72370	RPSAA	40s ribosomal protein SA
30	At1g55130	TMN6	Endomembrane protein 70 protein family
31	At3g55280	RPL23AB	ribosomal protein L23AB
32	At5g45590	L35	Ribosomal protein L35
33	At4g26300	emb1027	Arginyl-tRNA synthetase, class Ic
34	At1g72480	Lung seven transmembrane receptor	Lung seven transmembrane receptor family protein
35	At2g36620	RPL24A	ribosomal protein L24
36	At5g19750	Mpv17/PMP22	Peroxisomal membrane 22 kDa (Mpv17/PMP22) family protein
37	At3g55010	PUR5	phosphoribosylformylglycinamidine cyclo-ligase, chloroplast / phosphoribosyl-aminoimidazole synthetase / AIR synthase (PUR5)
38	At2g01720	Ribophorin I	Ribophorin I
39	At4g10480	Nascent polypeptide-associated complex (NAC),	Nascent polypeptide-associated complex (NAC), alpha subunit family protein
40	At3g51800	EBP1	metallopeptidase M24 family protein
41	At4g21150	HAP6	ribophorin II (RPN2) family protein
42	At5g64970	Mitochondrial substrate carrier	Mitochondrial substrate carrier family protein
43	At3g02530	chaperonin	TCP-1/cpn60 chaperonin family protein
44	At5g41000	YSL4	YELLOW STRIPE like 4
45	At3g59390		
46	At2g21160	Translocon-associated (TRAP),	Translocon-associated protein (TRAP), alpha subunit
47	At3g09200	L10	Ribosomal protein L10 family protein
48	At1g14320	SAC52	Ribosomal protein L16p/L10e family protein
49	At5g04060	transferase	S-adenosyl-L-methionine-dependent methyltransferases superfamily protein
50	At1g34130	STT3B	staurosporin and temperature sensitive 3-like b
51	At3g02650	Tetratricopeptide repeat (TPR)-like	Tetratricopeptide repeat (TPR)-like superfamily protein
52	At1g64790	ILITYHIA	ILITYHIA
53	At5g66680	DGL1	dolichyl-diphosphooligosaccharide-protein glycosyltransferase 48kDa subunit family protein
54	At3g09090	DEX1	defective in exine formation protein (DEX1)
55	At5g61170	S19e	Ribosomal protein S19e family protein
56	At3g18190	chaperonin	TCP-1/cpn60 chaperonin family protein
57	At3g48930	EMB1080	Nucleic acid-binding, OB-fold-like protein
58	At1g53240	mMDH1	Lactate/malate dehydrogenase family protein
59	At3g25040	ERD2B	endoplasmic reticulum retention defective 2B
60	At1g44835	tRNA syn	YbaK/aminoacyl-tRNA synthetase-associated domain
61	At1g77940	L7Ae/L30e/S12e/Gadd45	Ribosomal protein L7Ae/L30e/S12e/Gadd45 family protein
62	At3g47370	S10p/S20e	Ribosomal protein S10p/S20e family protein
63	At1g13730	NTF2	Nuclear transport factor 2 (NTF2) family protein with RNA binding (RRM-RBD-RNP motifs) domain
64	At5g02740	S24e	Ribosomal protein S24e family protein
65	At4g15000	L27e	Ribosomal L27e protein family
66	At1g24360	Rossmann-fold	NAD(P)-binding Rossmann-fold superfamily protein
67	At1g08350	Endomembrane 70	Endomembrane protein 70 protein family
68	At4g17390	L23/L15e	Ribosomal protein L23/L15e family protein
69	At1g12000	Phosphofructokinase	Phosphofructokinase family protein
70	At4g20980	eIF-3	Eukaryotic translation initiation factor 3 subunit 7 (eIF-3)
71	At3g16780	L19e	Ribosomal protein L19e family protein
72	At2g32060	L7Ae/L30e/S12e/Gadd45	Ribosomal protein L7Ae/L30e/S12e/Gadd45 family protein
73	At3g60830	ARP7	actin-related protein 7
74	At5g12470	DUF3411	Protein of unknown function (DUF3411)
75	At4g34555	S25	Ribosomal protein S25 family protein
76	At2g17630	transferase	Pyridoxal phosphate (PLP)-dependent transferases superfamily protein
77	At1g18540	Ribosomal L6	Ribosomal protein L6 family protein
78	At2g05755	transporter	Nodulin MtN21 /EamA-like transporter family protein
79	At5g17630	transporter	Nucleotide/sugar transporter family protein
80	At1g26910	RPL10B	Ribosomal protein L16p/L10e family protein
81	At1g26460	Tetratricopeptide repeat (TPR)-like	Tetratricopeptide repeat (TPR)-like superfamily protein
82	At5g06660	Protein of unknown function DUF106	Protein of unknown function DUF106, transmembrane
83	At1g07370	PCNA1	proliferating cellular nuclear antigen 1
84	At5g14030	TRAPB	translocon-associated protein beta (TRAPB) family protein
85	At3g03960	chaperonin	TCP-1/cpn60 chaperonin family protein
86	At5g15050	transferase	Core-2/I-branching beta-1,6-N-acetylglucosaminyltransferase family protein
87	At4g29120	dehydrogenase	6-phosphogluconate dehydrogenase family protein
88	At5g17250	Alkaline-phosphatase-like	Alkaline-phosphatase-like family protein
89	At5g25100	Endomembrane 70	Endomembrane protein 70 protein family
90	At3g07110	L13	Ribosomal protein L13 family protein
91	At3g60770	S13/S15	Ribosomal protein S13/S15
92	At4g33400	Vid27	Vacuolar import/degradation, Vid27-related protein
93	At3g20560	PDIL5-3	PDI-like 5-3
94	At1g18080	RACK1A_AT	Transducin/WD40 repeat-like superfamily protein
95	At3g02490	PPR	Pentatricopeptide repeat (PPR) superfamily protein
96	At5g10840	EMP1	Endomembrane protein 70 protein family
97	At5g64670	L18e/L15	Ribosomal protein L18e/L15 superfamily protein
98	At1g07070	L35Ae	Ribosomal protein L35Ae family protein
99	At2g30200	EMB3147	catalytics;transferases;[acyl-carrier-protein] S-malonyltransferases;binding
100	At5g02410	ALG10	DIE2/ALG10 family

Supplemental Table S5 Cre12g537641 Coexpressed Gene List.

ALCOdb	*C. reinhardtii*			*A.thaliana*	
Rank	Gene ID	Description	MR	Locus	Alias
Guide	Cre12g537641	[Ath: MBOAT (membrane bound O-acyl transferase) family protein]	0	At1g12640	LPLAT1
1	Cre14g615450	-	2.5	At5g54870	
2	Cre03g157800	Thioredoxin-like protein [Ath: atypical CYS HIS rich thioredoxin 2]	4	At3g03720	CAT4
3	Cre09g392579	-	5.7	At5g11960	DUF803
4	Cre14g627850	-	10.4	At3g17810	PYD1
5	Cre16g673550	[Ath: S-methyl-5-thioribose kinase]	10.6	At5g45410	
6	Cre08g377100	Adenylate kinase [Ath: adenosine kinase]	11	At3g09320	zinc finger
7	Cre17g711150	[Ath: fatty acid desaturase 2]	13.4	At2g02040	PTR2-B
8	Cre10g434250	[Ath: translocase of inner mitochondrial membrane 23]	16.9	At3g51730	saposin B
9	Cre06g254600	-	17.7	At3g02740	protease
10	Cre13g571000	-	17.8	At3g63310	BIL4
11	Cre12g556600	Glyceraldehyde 3-phosphate dehydrogenase, nonphosphorylating [Ath: aldehyde dehydrogenase 11A3]	18.3	At3g04090	SIP1A
12	Cre07g348600	Chloroplast sulfate permease	19.3	At4g19640	RABF2B
13	Cre09g404450	[Ath: Mitochondrial substrate carrier family protein]	22.8	At1g04970	glycoprotein
14	Cre10g422300	Thioredoxin dependent peroxidase [Ath: Thioredoxin superfamily protein]	23.8	At3g19950	box
15	Cre06g279150	[Ath: Class II aminoacyl-tRNA and biotin synthetases superfamily protein]	26.9	At3g51130	
16	Cre10g461900	[Ath: NAD(P)-linked oxidoreductase superfamily protein]	27.1	At1g54710	G18H
17	Cre14g620300	Anthranilate synthase, beta subunit	28.8	At5g01800	saposin B
18	Cre07g324200	Diacylglyceryl-N,N,N-trimethylhomoserine synthesis protein	28.9	At1g23440	I-like
19	Cre08g364450	Peptide alpha-N-acetyltransferase [Ath: Acyl-CoA N-acyltransferases (NAT) superfamily protein]	31	At5g51710	KEA5
20	Cre03g189400	Seryl-tRNA(Sec) synthetase [Ath: seryl-tRNA synthetase / serine--tRNA ligase]	31.8	At3g58750	CSY2
21	Cre09g393200	Heat shock protein 70C [Ath: mitochondrial HSO70 2]	33.5	At3g25585	AAPT2
22	Cre01g033400	Mitochondrial inner membrane translocase [Ath: Tim10/DDP family zinc finger protein]	34.2	At1g32790	CID11
23	Cre17g717250	-	34.4	At1g02816	DUF538
24	Cre15g641700	-	34.6	At5g07220	BAG3
25	Cre12g533351	[Ath: heat shock protein 101]	38.1	At5g02040	PRA1.A1
26	Cre03g193800	-	38.9	At3g61070	PEX11E
27	Cre13g568600	[Ath: histidine acid phosphatase family protein]	39.4	At4g28770	Tetraspanin
28	Cre10g427700	[Ath: P-loop containing nucleoside triphosphate hydrolases superfamily protein]	41.8	At3g26100	RCC1
29	Cre11g480650	-	42.1	At2g02870	oxidase/kelc
30	Cre03g144967	[Ath: P-loop containing nucleoside triphosphate hydrolases superfamily protein]	43.1	At3g30390	transporter
31	Cre10g424450	40 kDa translocon at mitochondrial outer envelope membrane [Ath: translocase of the outer mitochondrial membrane 40]	43.4	At1g16180	domain
32	Cre10g450150	-	45.5	At4g39140	box
33	Cre11g482750	-	46.5	At1g77770	DUF1644
34	Cre06g302750	[Ath: Transducin/WD40 repeat-like superfamily protein]	47	At3g56310	Melibiase
35	Cre10g452650	Mitochondrial inner membrane translocase [Ath: translocase inner membrane subunit 17-2]	48.6	At4g16110	RR2
36	Cre06g306601	[Ath: anthranilate synthase alpha subunit 1]	49.6	At5g59840	binding
37	Cre07g337800	Mitochondrial ribosomal protein S17 [Ath: Nucleic acid-binding, OB-fold-like protein]	49.8	At2g31350	GLX2-5
38	Cre12g551500	DnaJ-like protein [Ath: gametophytic factor 2]	49.9	At1g68000	PIS1
39	Cre02g074100	-	53.5	At2g39780	RNS2
40	Cre10g435500	Elongation factor Ts-like protein [Ath: translation elongation factor Ts (EF-Ts), putative]	53.6	At5g27150	NHX1
41	Cre05g242950	Putative mitochondrial ribosomal protein L22, imported to mitochondria	55	At1g71190	SAG18
42	Cre11g468350	-	55.6	At2g43790	MPK6
43	Cre13g580150	[Ath: Major facilitator superfamily protein]	55.7	At3g17000	UBC32
44	Cre06g291150	[Ath: DEAD box RNA helicase family protein]	56.5	At1g47128	RD21A
45	Cre11g467553	-	56.5	At5g49890	CLC-C
46	Cre17g699900	[Ath: NAD(P)-linked oxidoreductase superfamily protein]	56.6	At4g32680	
47	Cre17g712000	Serine/arginine-rich pre-mRNA splicing factor [Ath: RS-containing zinc finger protein 21]	59.3	At1g29330	ERD2
48	Cre09g395621	[Ath: 2A phosphatase associated protein of 46 kD]	60.3	At2g28110	IRX7
49	Cre06g303171	-	60.6	At4g21160	ZAC
50	Cre17g726400	[Ath: LAG1 homologue 2]	61.8	At5g64930	HYS1
51	Cre01g002100	-	62.2	At1g23040	glycoprotein
52	Cre06g275300	-	62.6	At5g42090	transmembra
53	Cre03g209617	-	62.9	At4g35860	GB2
54	Cre10g457700	[Ath: calmodulin-domain protein kinase cdpk isoform 2]	63.5	At5g54080	HGO
55	Cre14g614950	Putative mitochondrial ribosomal protein S2, imported to mitochondria	63.6	At1g02305	Cysteineases
56	Cre09g397900	[Ath: Transmembrane CLPTM1 family protein]	64.1	At4g14950	KMS1
57	Cre13g587050	Eukaryotic release factor	65.6	At2g01540	dependent
58	Cre03g145727	-	67.7	At4g16760	ACX1
59	Cre12g553550	-	69.2	At1g25520	UPF0016
60	Cre01g034050	[Ath: Pyridoxal phosphate (PLP)-dependent transferases superfamily protein]	75.6	At5g44790	RAN1
61	Cre03g156350	-	76.5	At3g43230	zinc finger
62	Cre16g657850	ER-targeted preprotein translocase subunit [Ath: DnaJ / Sec63 Brl domains-containing protein]	77.6	At1g74020	SS2
63	Cre09g412300	Presequence translocase-associated protein import motor subunit [Ath: Protein Transporter, Pam16]	77.8	At2g41490	GPT
64	Cre14g615850	-	77.9	At4g21980	G8A
65	Cre03g182500	Subunit of the Signal Recognition Particle	79	At3g11900	ANT1
66	Cre05g245102	-	79.4	At3g22290	transporter
67	Cre06g278210	Phosphoglucomutase [Ath: phosphoglucomutase]	80.4	At4g13270	LEA
68	Cre17g738350	-	80.4	At1g75760	retaining
69	Cre06g309100	Chaperonin 60C [Ath: heat shock protein 60]	83.1	At5g45900	ATG7
70	Cre03g170450	-	84.3	At5g35460	
71	Cre16g667150	-	84.8	At1g29760	Seipin
72	Cre16g677000	Heat shock protein 70E	84.8	At2g17500	carrier
73	Cre06g278202	[Ath: Inositol monophosphatase family protein]	85.4	At5g61640	PMSR1
74	Cre11g467575	[Ath: casein lytic proteinase B3]	85.9	At5g64370	PYD3
75	Cre03g180300	Sulfite reductase [Ath: P450 reductase 2]	86	At3g23560	ALF5
76	Cre12g521650	[Ath: Esterase/lipase/thioesterase family protein]	86.5	At4g31130	DUF1218
77	Cre14g618200	-	87.6	At2g23310	RER1C1
78	Cre11g467702	-	87.7	At1g53580	GLY3
79	Cre13g578900	-	88.7	At5g24870	box
80	Cre02g117300	Putative mitochondrial ribosomal protein L45, imported to mitochondria [Ath: Mitochondrial inner membrane translocase complex, subunit Tim44-related protein]	88.7	At3g54680	hoglycan
81	Cre16g690050	Cytochrome c heme lyase	89.2	At3g49210	WSD1-like
82	Cre10g428150	[Ath: ABC2 homolog 9]	89.6	At3g53780	RBL4
83	Cre14g615950	[Ath: general control non-repressible 4]	91.8	At3g51990	kinase
84	Cre01g022250	Mitochondrial ribosomal protein L3 [Ath: ribosomal protein L3 plastid]	92.2	At5g58730	Mik
85	Cre13g576760	[Ath: Chlorophyll A-B binding family protein]	93.4	At2g28370	UPF0497
86	Cre14g618700	Mitochondrial ribosomal protein S7	94.1	At4g27320	PHOS34
87	Cre12g544050	[Ath: poly(A) binding protein 8]	94.9	At2g39970	PXN
88	Cre16g651750	-	95.3	At4g27780	ACBP2
89	Cre02g108200	Predicted protein	95.4	At1g28960	NUDX15
90	Cre02g091450	-	98	At4g40050	PF0682
91	Cre06g278167	-	98.1	At5g58060	YKT61
92	Cre16g656400	UDP-sulfoquinovose synthase [Ath: sulfoquinovosyldiacylglycerol 1]	99.1	At5g40470	RNI-like
93	Cre05g241639	-	99.6	At2g42790	CSY3
94	Cre01g036950	-	101	At5g57740	XBAT32
95	Cre12g555500	-	102	At1g29970	RPL18AA
96	Cre17g722800	Mitochondrial processing peptidase alpha subunit [Ath: Insulinase (Peptidase family M16) protein]	103	At4g17530	RAB1C
97	Cre12g558650	Eukaryotic translation initiation factor 2B subunit 4, eIF-2B delta subunit	104	At5g45550	MOB1-like
98	Cre06g278225	[Ath: tetratricopeptide repeat (TPR)-containing protein]	104	At1g60430	ARPC3
99	Cre13g603900	[Ath: tRNA synthetase beta subunit family protein]	104	At4g11220	RTNLB2
100	Cre10g443700	-	104	At5g18900	2OG

MR, matual rank. smaller MR indicates stronger coexpression.

[Authors: Please check the definition of "MR". Should this be "mutual"?]

Supplemental Table S6 The mass of different MGMG species monitored in MRM mode.

Molecular species	Precursor ion $[M+NH_4]^+$ mass	Product ion mass
C16:0-MGMG	510.4	331.3
C16:1-MGMG	508.4	329.3
C16:2-MGMG	506.3	327.3
C16:3-MGMG	504.3	325.3
C16:4-MGMG	502.3	323.2

MRM, multiple reaction monitoring

The retention time for MGMG species was 4-10 min.

Supplemental Table S7 Primer sequences used in this study.

Primer	Orientation	Sequence (5'–3')
CrLAT1 for semi-quantitative PCR in Figure 4	Forward	CCATCGTCGACGAGGTGGCAAAG
	Reverse	CGGCACACGCAGATGGAGATGAG
CBLP for semi-quantitative PCR in Figure 4	Forward	GTGCAGGACGTGGTCATCTC
	Reverse	GTCACGGTGTTGACGTAGCC
Primer 1 in Figure 5	Forward	AGGCGGCGAGTATGAGTAGA
Primer 2 in Figure 5	Forward	GACGTTACAGCACACCCTTG
Primer 3 in Figure 5	Reverse	ACATCCCTTCCCAGTCACCC
CrLAT1 for quantitative PCR in Figure 5, Supplemental Figure 6	Forward	ACCACCTTCGTGCTCAACTACA
	Reverse	CGTTCACCACAAGCATCACC
CBLP for quantitative PCR in Figure 5, Supplemental Figure 6	Forward	TGCTGTCCGTGGCTTTCTC
	Reverse	GGCTCGCCAATGGTGTACTT
CrLAT1 overexpression primer 1	Forward	CTGCAGATGGAGCAGGTGGAGGCACAG
CrLAT1 overexpression primer 2	Reverse	GAATTCTCACTCGGCCTTGACGCCATTC
